# Rapid, modular, and cost-effective generation of donor DNA constructs for CRISPR-based gene knock-in

**DOI:** 10.1093/biomethods/bpaa006

**Published:** 2020-03-20

**Authors:** Yi-Jiun Chen, Ya-Yun Cheng, Weikang Wang, Xiao-Jun Tian, Daniel E Lefever, David A Taft, Jingyu Zhang, Jianhua Xing

**Affiliations:** b1 Department of Computational and Systems Biology, School of Medicine, University of Pittsburgh, Pittsburgh, PA 15260, USA; b2 Drug Discovery Institute, School of Medicine, University of Pittsburgh, Pittsburgh, PA 15260, USA

**Keywords:** CRISPR knock-in, donor construct, in silico screening, molecular cloning

## Abstract

Clustered regularly interspaced short palindromic repeats (CRISPR)-based gene editing techniques find applications in many fields, such as molecular biology, cancer biology, and disease modeling. In contrast to the knock-out procedure, a key step of CRISPR knock-in experiments is the homology-directed repair process that requires donor constructs as repair templates. Therefore, it is desirable to generate a series of donor templates efficiently and cost-effectively. In this study, we developed a new strategy that combines (i) Gibson assembly reaction, (ii) a linker pair composed of eight in silico screened restriction enzyme sites, and (iii) a hierarchical framework, to remarkably improve the efficiency of producing donor constructs for common genes as well as for the genes containing unbalanced guanine-cytosine content and requiring a selectable marker. Furthermore, the approach provides the ability of inserting additional elements into the donor templates, such as single guide RNA recognition sites that have been reported to enhance the efficiency of homology-directed repair. Conclusively, our modularized process is simple, fast, and cost-effective for making donor constructs and benefits the application of CRISPR knock-in methods.

## Introduction

In a cell, there are multiple signaling pathways that respond to intracellular and extracellular signals, thereby regulating gene expression. To investigate mechanisms of signal transduction, it is desirable to generate a series of knock-in mutants fused with fluorescence proteins (FPs) for tracking protein dynamics [1, 2], and CRISPR knock-in is a method of choice. Unlike knocking out genes, a CRISPR knock-in process [3–5] requires additional donor DNA containing the specific knock-in sequences and the repair templates, exploiting the mechanism of homology-directed repair (HDR) [6]. In general, the knock-in procedure is not trivial. One challenge is to obtain high HDR efficiency for CRISPR-based gene editing. Some studies have shown that DNA nicks [7], suppression of KU70 and DNA ligase IV [8], and a donor template, in which the inserted fragments (IFs) flanked by single guide RNA (sgRNA) recognition sites [9], improve the experiment outcome. Another challenge is producing donor DNA for multiple target genes in an efficient and cost-effective way.

A typical donor construct has four major components: the vector backbone, the IF, such as an FP, and the two homologous fragments (5’ and 3’ arms) localized at the two sides of the insertion point. Mainly, there are two categories of methods for assembling these components into a complete donor construct: restriction enzyme (RE)-based approaches and sequence-independent overlapping techniques. Examples for the first category are BioBricks [10], BglBricks [11], and Golden Gate [12], while for the latter, Circular Polymerase Extension Cloning [13], Sequence-Ligation Independent Cloning [14], Overlap Extension Polymerase Chain Reaction (PCR) [15], and Gibson isothermal assembly [16, 17] are commonly mentioned. Each assembly standard contains pros and cons [18, 19]; thus, researchers choose the appropriate approaches according to the feature of the genes cloned, for instance, the complexity and the length of the sequences. Among these methods, Gibson assembly is one of the most well-accepted and widely used techniques in labs [20]. It is rapid and convenient because multiple fragments are assembled in a defined order within a single-tube isothermal reaction. However, there are some drawbacks to such a sequence-independent overlapping standard. Gibson assembly is not suitable for assembling sequences harboring many repeats, high guanine-cytosine (GC) content, or potential secondary structure [21], from which researchers usually obtain misassembled products.

Given the need of generating donor constructs more efficiently and cost-effectively, we established a new strategy that combines Gibson assembly, the Modular Overlap-Directed Assembly with Linkers (MODAL) standard [22], and the hierarchical framework approach [23] to overcome the limitation of Gibson assembly, to enhance the reaction efficiency, and to add additional assembly possibility to the constructs for the potential requirements in the future experiments. MODAL uses in silico screening to design optimal linker sequences, which serves as the overlapping sequences between DNA fragments for guiding assembly and allows the usage for repeat or high GC content fragments. Meanwhile, the hierarchical framework integrates RE digestion and Gibson assembly, enabling the construction of synthetic gene circuits with large sizes. In this study, we created a new pipeline for screening the proper restriction enzyme site (RES) sequences that appear only a few times in the human genome and applied them as the linkers for Gibson assembly reaction as well as the subsequent digestion–ligation steps if needed.

Our method incorporates the advantages of the RE-based approaches and sequence-independent overlapping techniques. On one hand, the assembly step is based on Gibson reaction, for which we introduced the linkers to cope with the constraints and to increase the efficiency up to eight-fold. On the other hand, the RESs employed here can be easily used for subsequent experiments. We demonstrated that, the HDR efficiency was improved to approximately two-fold by inserting the sgRNA recognition sites, which comprise the sgRNA target sequences and the protospacer adjacent motif (PAM) sequences (5'-NGG-3'), to our IF vector through a simple digestion–ligation step. In our group, we already created the donor vector bank where the donor vectors with various reporter proteins (RPs), FPs, and/or removable selectable markers (SMs), flanked by the linkers on standby. Depending on the different experimental purposes, the corresponding IF vector is chosen to perform one digestion plus one assembly reaction, and then the donor template is ready. Overall, the new approach and our donor vector bank facilitate the production of donor constructs required for CRISPR knock-in method, hence expanding the application of CRISPR-based techniques to a broader range of genes, discovering the unknown functions and mechanisms in cells.

## Material and methods

### In silico screening of the linkers

For generating donor DNA, we introduced a pair of linkers (a and b) into Gibson assembly reaction. The linkers were composed of specific RESs, with *Age*I site at the 5’ end of linker a, *Xho*I site at the 3’ end of linker b, and three other RESs on each linker (See Supplementary data and Supplementary Table S1). The linkers are needed for reducing the error rate of PCR amplification, increasing modularity of the procedure, and applying the hierarchical framework [23]. We developed a computer program to select optimal linker sequences. First, a list for all possible sequences with the length of 30 base pairs (bps) was produced and subjected to in silico screening to remove the ones harboring adverse factors for PCR reaction, such as single-stranded DNA secondary structures, potential of dimerization, high salt-adjusted melting temperature, unbalanced GC content, nucleotide repeats, and GC clamp. Next, we performed Basic Local Alignment Search Tool (BLAST) to pick up the candidates with the lowest identity to the human genomic library (Supplementary Fig. S1). Through the overall screening procedure, we identified 12 pairs of linkers (Supplementary Table S2) and selected two pairs, ranked numbers 1 and 6, for the subsequent experimental tests. See Supplementary Data for a detailed description of the screening.

### Cloning and PCR methods

The FP vector was purchased from Addgene (pcDNA3-EGFP, #13031) and the linker a and b were inserted, therefore becoming the IF vector used in Fig. 2A. The mCherry construct was kindly provided by Dr. Robin E. C. Lee. For testing how LoxP sites affect assembly efficiency, we amplified the fragments of Enhanced Green Fluorescent Protein (EGFP), EGFP with two flanking LoxP sites (LoxP-EGFP-LoxP), SM (neomycin-resistance gene), SM with two flanking LoxP sites (LoxP-SM-LoxP), SM-EGFP, and SM-EGFP with two flanking LoxP sites (LoxP-SM-LoxP-EGFP) and inserted them separately into pcDNA3 vector with or without linker pair 1. Additionally, we also amplified the mCherry with LoxP-Neomycin-LoxP and inserted it into the pcDNA3 vector to form the IF vector mentioned in Fig. 4A. As for the donor template generation, the homologous arms of the knock-in target genes were obtained by PCR and then the DNA fragments as well as the IF vectors were subjected to RE digestion. All the primers used in this study are listed in Supplementary Table S3. The genomic DNA from T47D cells (ATCC#HTB-133) was extracted by QIAGEN DNeasy Blood & Tissue Kit (#69504). The PCR experiments were performed with Q5 High-Fidelity 2X Master Mix (NEB#M0492). The amplified DNA fragments were purified by GeneJET PCR Purification Kit (Thermo#K0702) or GeneJET Gel Extraction Kit (Thermo#K0692). The REs were purchased from New England Biolabs (NEB). Subsequently, the digested products were assembled by Gibson assembly reaction (NEB#E2611) and proceeded to transformation. The next day, we picked up colonies and grew them in LB medium with 50 µg/ml ampicillin. Afterward, the GeneJET Plasmid Miniprep Kit (Thermo#K0503) was used for plasmid purifications. To verify the donor constructs by PCR, we chose regular GoTaqG2 Flexi DNA polymerase (Promega#M7805) and the Deoxynucleotide Solution Set (dNTP, NEB#N0446).

### Assembly efficiency calculation

The digested DNA fragments and vectors were purified and then mixed with NEBuilder HiFi DNA Assembly Master Mix (NEB#E2621), incubating for 1–3 hours. The DNA assembly mixtures were transformed into DH5α cells. The next day, 60 colonies were picked for PCR verification to check the IFs. The efficiency was calculated as the number of the colonies containing the desirable constructs divided by the number of tested colonies.

### GC content calculation

The human reference genome (GRCh38/hg38) was downloaded from the National Center for Biotechnology Information database (GCF_000001405.34_GRCh38.p8_genomic.gbff), and split into different GenBank files for every chromosome as well as one for mitochondrial DNA. Analysis was performed on each chromosome individually. Alternate assemblies and unassembled reads were not included in the analysis. A combination of homebrewed scripts and Biopython [24] was used to parse the individual GenBank files and then extract the coding domain sequence (CDS) features for every file. For every CDS feature, the regions of ±750 bps from the start and stop codons were extracted. Then, the percentage of GC content was calculated by: (Σ*G* + Σ*C*)/Σ*N* × 100%, where *N* represents for any type of the nucleobases. The results were subsequently turned into a list. Since there are potentially multiple CDS entries for a given gene, with different start/stop positions, the list was filtered according to the condition that only one CDS entry for a unique start/stop position was included. A command-line script is provided as a Supplementary data named *chromo_gc_content.py* [requirement: Python 2.7 and python package Biopython (http://biopython.org/)].

### Cell culture and knock-in efficiency calculation

T47D cells (ATCC#HTB-133) were cultured in Dulbecco’s Modified Eagle’s Medium (Gibco) supplemented with 10% fetal bovine serum (Biochrom), 100 units/ml penicillin and 100 µg/ml streptomycin (Invitrogen) in 5% CO_2_ at 37°C. For transfection, the cells were seeded on 35 mm dishes one day before the experiments. The next day, 750 ng of the donor template and 750 ng of the sgRNA-Cas9 plasmid were co-transfected into the cells, using FuGENE HD Transfection Reagent (Promega #E2311). Afterward, the cells were incubated for 48 hours and subcultured in 10 cm dishes for 5–7 days before subjected to image-taking or knock-in efficiency assay. The sgRNA sequence of *CDH1* is 5′-AAGCTGGCTGACATGTACGG-3′, which was designed by the program named “CRISPR gRNA Design tool – ATUM” (https://www.atum.bio/eCommerce/cas9/input). For the sequences of the sgRNA recognition site, the PAM sequences were added to the 3’ end, marked in bold: 5′-AAGCTGGCTGACATGTACGG**AGG**-3′. After annealing the two single-stranded oligonucleotides, the sgRNA-PAM fragments were inserted into the donor construct. Through flow cytometry, the knock-in efficiency was calculated as the number of EGFP-positive cells divided by the total number of the cells.

## Results

### Twelve universal pairs of linkers were selected through in silico screening

For generating CRISPR knock-in donor constructs, Gibson assembly is the method of choice to join the vector backbone, the IF, and the two homologous fragments (5’ and 3’ arms) up. DNA synthesis is a straightforward and fast way to obtain the 5’ and 3’ homologous arms of target genes. However, this approach is only suitable for low complexity fragments with few repeat sequences and homopolymeric regions, and without unbalanced GC content [25]. Alternatively, the homologous arms can be amplified from human genome by PCR. For the present purpose, two elements are needed in the PCR primers: the annealing sequences of the target genes for amplifying the homologous arms and the overlapping sequences between the DNA fragments to direct the assembly reaction in a determined order [19]. After getting the synthesized or amplified products, Gibson assembly is performed to construct the complete donor template. Nevertheless, the assembly reaction is, in general, not efficient enough, and for the sequences with high GC content or repeats, it usually results in a misassembled outcome. Therefore, to overcome the limitation of Gibson assembly, it is desirable to have a new approach providing higher accuracy and efficiency. In this study, we developed a computational pipeline to choose suitable RE recognition sequences, using them as the linkers for improving Gibson assembly reaction and inserting additional fragments when it is needed.

**Figure 1:**
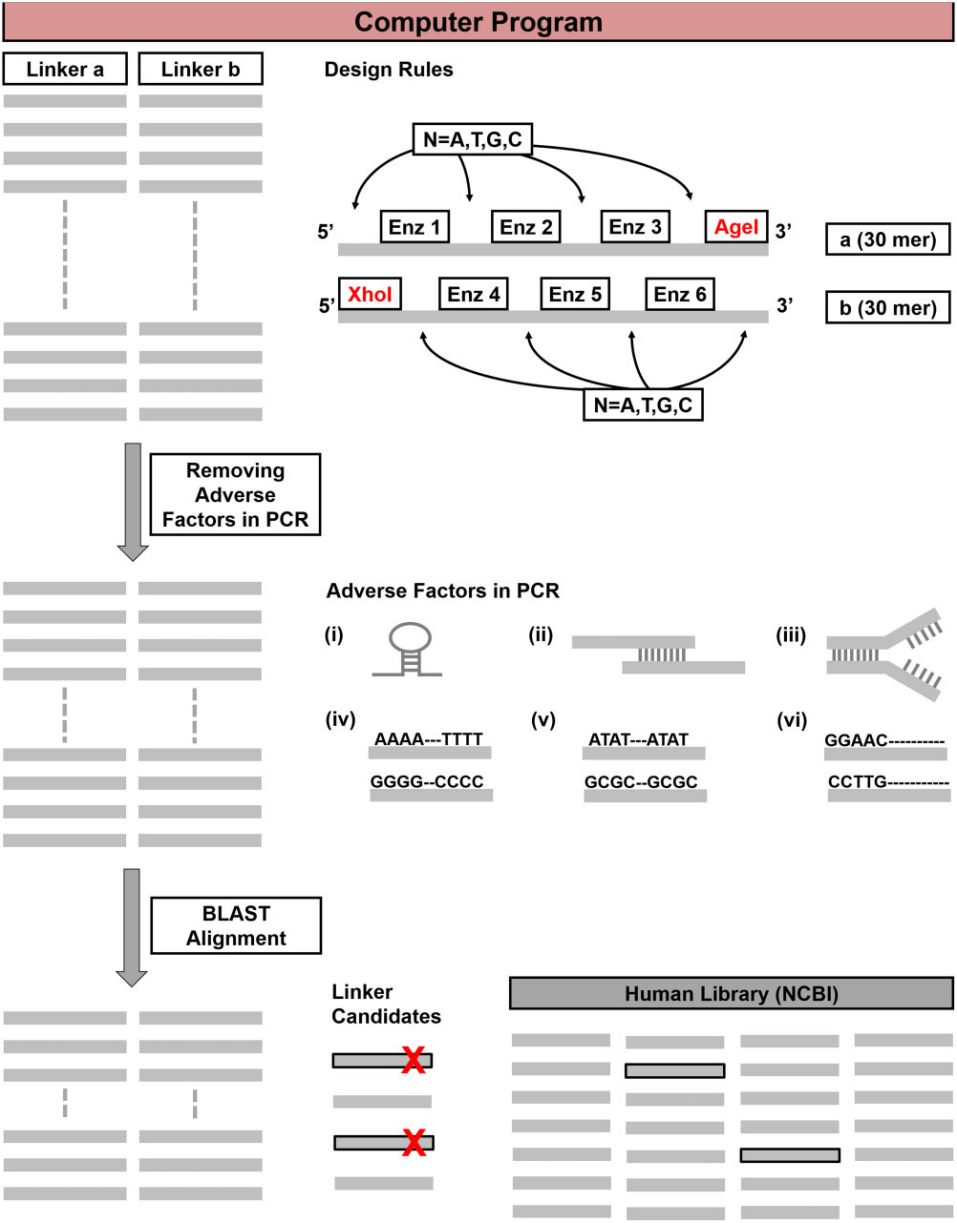
In silico screening of the linkers. We developed a computational pipeline to design the linker pairs with the length of 30 bps, containing specific RESs. For a linker pair (linkers a and b), there were total eight RESs assigned. *Age*I site was at the 5’ end of linker a, XhoI was at the 3’ end of linker b, and six other RESs (Enzs 1–6) in the middle. “N” represents any nucleotides localized between the RESs for filling up the spaces and creating the probability of the sequence combinations for the linkers. According to our concept, as the first step, the computer program generated a list of all the possible linker pair sequences. Next, the sequences that harbor adverse factors for PCR reaction were removed. Examples for adverse factors are (i) single-stranded DNA secondary structures, such as hairpin, (ii) potential of dimerization, (iii) high salt-adjusted melting temperature, (iv) unbalanced GC content, (v) nucleotide repeats, and (vi) GC clamp. Last, the BLAST alignment step was applied to further filter the candidates and select the linker pairs with low identity to the human genome.

Our idea came from the MODAL strategy that applied linkers to conduct Gibson assembly [22]. However, the study was demonstrated for bacterial or yeast genome. To make it more practical for investigating human genes, we selected optimal sequences based on human genome. With the aim of picking the best linker sequences, we created a computer program (Fig. 1) to design sequences with a length of 30 bps, in which four RESs were assigned, and subjected all possible results to in silico screening to remove adverse factors for PCR reaction, for instance, single-stranded DNA secondary structures, potential of self/cross-dimerization, high salt-adjusted melting temperature, unbalanced GC content, nucleotide repeats, and GC clamp. Next, to minimize sequence identity between the linkers and the human genome, a BLAST alignment step was set to further filter the remaining candidates (Supplementary Fig. S1). According to the screening pipeline, we identified 12 pairs of linkers (Supplementary Table S2) and selected the top- as well as the middle-ranked linker pairs (1 and 6) for the subsequent experimental tests. Finally, the PCR primers were designed with a total length of 60 bps, including 30 bps of annealing sequences for amplifying the target gene and 30 bps linkers as the overlapping regions for guiding Gibson assembly reaction.

### The linkers increased Gibson assembly efficiency for generating knock-in donor constructs

**Figure 2:**
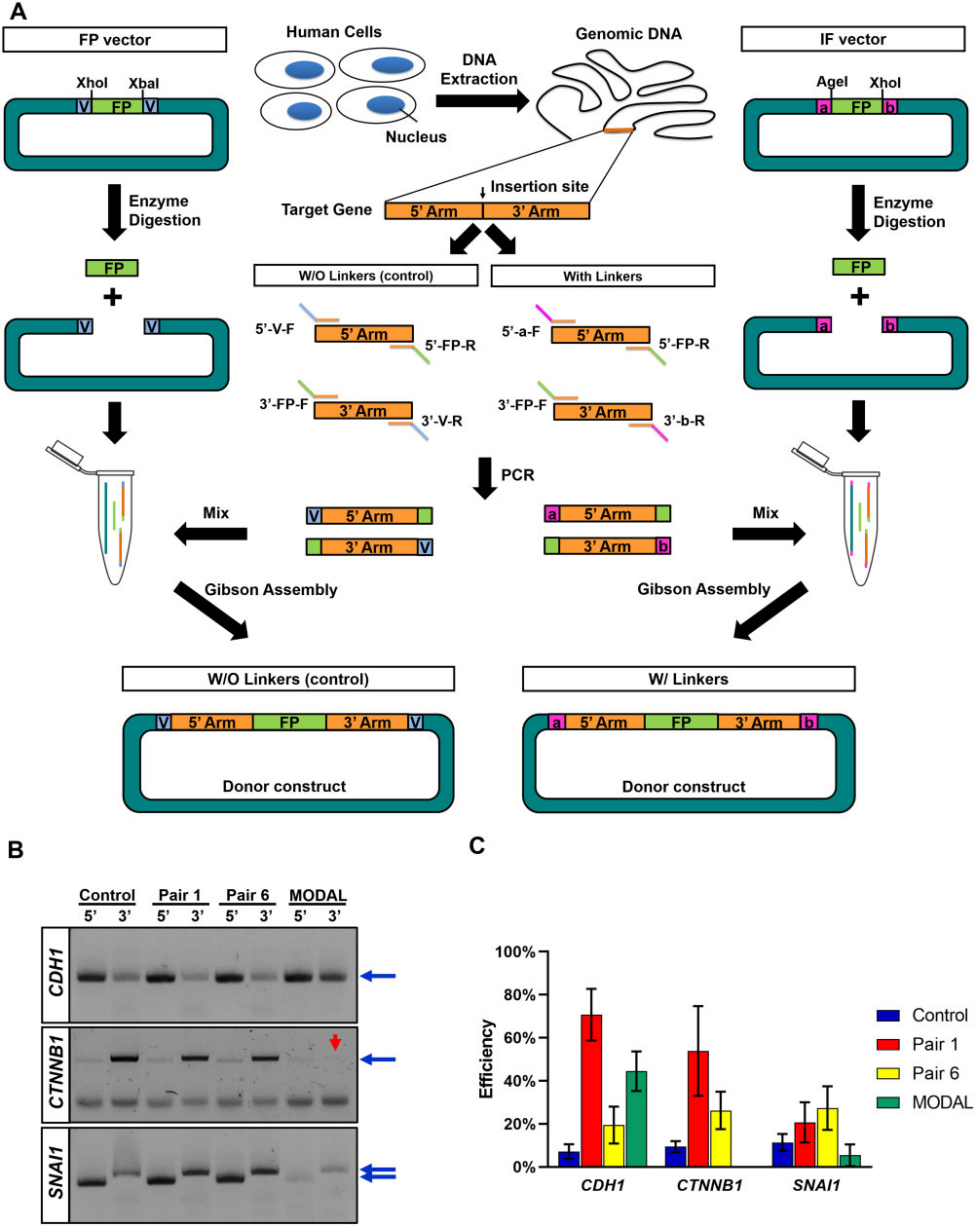
The in silico selected linker pairs improved the efficiency of Gibson assembly reaction. (**A**) Schematic diagram of donor constructs production for the negative control, FP vector (left), and for the IF vector containing linker a and b (right). a: linker a, b: linker b, V: linker negative control, vector sequences. (**B**) The PCR results for the 5’/3’ homologous arm of the target genes. The linker pairs 1 and 6 were included in the primers for verification, while the original MODAL linker pair was used as the positive control. For the negative control, the linker sequences were replaced by the sequences from the vector itself. Blue arrows indicate the correctly amplified DNA fragments from *CDH1*, *CTNNB1*, and *SNAI1*. Red arrow points out the failed amplification for *CTNNB1* by the MODAL linker incorporated primers. (**C**) The efficiency of Gibson assembly reaction. The efficiency was calculated as the number of the colonies with the desirable constructs divided by the number of tested colonies. The experiments were conducted in triplicate. The error bars indicate mean ± SD.

In order to verify our approach in the real-world CRISPR knock-in practice, we chose genes involved in the epithelial-to-mesenchymal transition (EMT) [26–29] as a model, since endogenously FP insertion allows live-cell time-lapse tracking of the spatial–temporal dynamics, which is an important technique to study cellular morphology changes and the mechanisms of EMT. First, we produced a set of vectors harboring the linkers and FP sequences to serve as the IF vectors (Fig. 2A, right). For the purpose of verification, linker pairs 1 and 6 obtained from our in silico screening were employed, with EGFP as the inserted FP, in the IF vectors. Meanwhile, we replaced the linker sequences with the sequences from the vector itself to be the FP vector and used it as the negative control (Fig. 2A, left). Additionally, the vector containing FP sequences and MODAL linkers generated by the R2oDNA Designer program [22] was included as the positive control (Supplementary Table S2).

Next, three EMT-related genes, *CDH1* (coding E-cadherin), *CTNNB1* (coding β-catenin), and *SNAI1* (coding Snail1), were picked as examples to test our method for producing donor constructs. Here, we aimed at inserting EGFP to the 3’ end of *CDH1*, the 5’ end of *CTNNB1*, and the 5’ end of *SNAI1*. To do so, we designed primers to amplify the homologous arms from human genomic DNA and attach the linker sequences at the same time by PCR. Consequently, the results showed that all the 5’ and 3’ arms of the three genes, except the 3’ arm of *CTNNB1* with MODAL linkers (red arrow), were successfully amplified by the designed primers (Fig. 2B). Afterward, the PCR products and the IF/FP vectors that were linearized through the RESs on the linkers were proceeded to Gibson assembly reaction. We performed PCR to verify the colonies and 10 clones from each sample were sent for sequencing (Supplementary Table S4 and Supplementary Fig. S2). The assembly efficiency was calculated by dividing the number of correct colonies by the number of tested colonies. Compared to the negative control, the presence of the linkers resulted in higher Gibson assembly efficiency (Fig. 2C). For *CDH1*, the linker pairs 1, 6, and MODAL increased the efficiency to nearly eight-, three-, and six-fold, respectively. Regarding *CTNNB1*, the linker pairs 1 and 6 led to approximately six- and three-fold efficiency improvement. The effect of the MODAL linkers was not shown because the corresponding primers were not able to amplify the DNA fragments by PCR. As for *SNAI1*, both the linker pairs 1 and 6 caused roughly two-fold higher efficiency. Altogether, we proved that introducing the linkers that were in silico selected by our computational workflow to the assembly reaction facilitates donor construct generation, thereby lowering the difficulty of performing CRISPR knock-in techniques.

### Unbalanced GC content of homologous fragments and removable SM severely reduced the Gibson assembly efficiency for generating donor constructs

**Figure 3:**
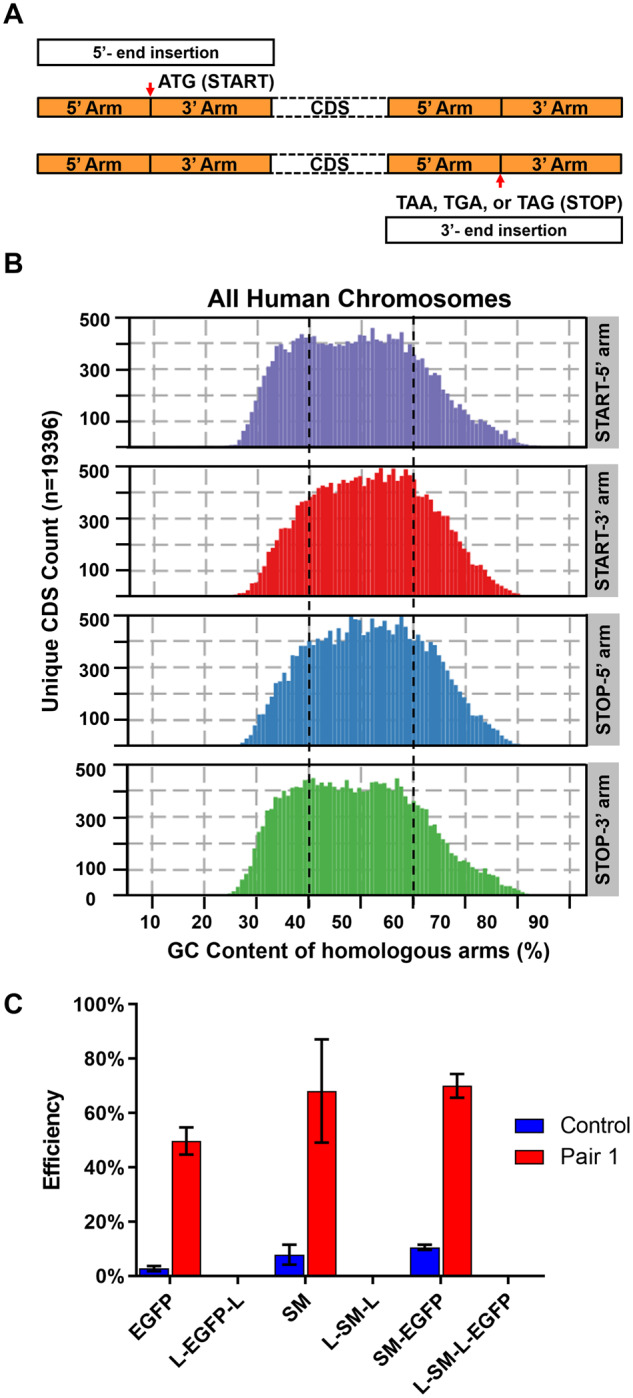
Unbalanced GC content and removable SM were the unfavorable factors affecting Gibson assembly. (**A**) The outline of the insertion sites for a target gene. The red arrows show the insertion positions of a gene. (**B**) The genome-wide distribution of the GC content for the 750 bps sequences at both sides of the start/stop codon. The unbalanced GC content is defined as higher than 60% or lower than 40% (black dashed lines). (**C**) The efficiency of Gibson assembly reaction for the IF with/without LoxP sites. Three different IFs, EGFP, SM and SM-EGFP were assembled with the homologous arms of *CDH1* and the IF vector. For the IF vector, the control vector and the one containing linker pair 1 were applied. The efficiency was calculated as the number of the colonies with the desirable constructs divided by the number of tested colonies. The experiments were conducted in triplicate. The error bars indicate mean ± SD. L, LoxP.

While confirming the effectiveness of our new method, we noticed that two factors, unbalanced GC content and removable SM, negatively influence Gibson assembly reaction. According to the results shown in Fig. 2C, the efficiency of Gibson assembly for *SNAI1* is, in general, lower than *CDH1* and *CTNNB1*. After checking the sequences of the 5’ and 3’ homologous arms, we found that *SNAI1* contains much higher GC content (72.8% and 60.5%) than *CDH1* and *CTNNB1* (Supplementary Table S5). The data implied that the reduced assembly efficiency was caused by the high GC content. Considering that a knock-in insertion site is restrictedly chosen close to either the start or stop codon of a target gene (Fig. 3A), we analyzed the whole human genome, calculating the GC content of the 750 bps regions flanking the start and stop codons. The results revealed that up to 45% of human genes contain unbalanced GC content (>60% or <40%) at one or both flanking regions, which make the donor construct production more challenging (Fig. 3B). Another difficulty one may face when performing CRISPR knock-in experiments is producing donor constructs for genes with low expression. For such silent genes, adding a removable SM (LoxP-SM-LoxP) driven by an external promoter in the donor template is necessary for screening successfully edited cells, since the genes themselves hardly express under normal cellular conditions. Then, the SM is removed after selection (Supplementary Fig. S3). Based on our experiences, including a removable SM in the procedure of generating donor templates hindered the assembly reaction. While performing Gibson assembly with the IF containing removable SM, we usually obtained very few colonies and the efficiency was considerably low.

Due to the problems we have encountered, we wondered whether our approach improves producing the donor templates with a removable SM for the genes carrying unbalanced GC content. Interestingly, *SNAI1* and *VIM* are the examples of genes that have low expression in epithelial cells [30] and unbalanced GC content near the start/stop codon (Supplementary Table S5); therefore, we took these two genes for further verification. First, the IF vectors harboring the linker pair 1, removable SM, and the FP (mCherry) were made. The Neomycin-resistance gene, flanked by two LoxP sites, was placed after (Supplementary Fig. S4A, left) or before the FP (Supplementary Fig. S4A, right). Then, the Gibson assembly reaction was conducted to incorporate the 5’ and 3’ homologous arms of the target genes. Consequently, compared to the outcome of the assembly reaction performed in Fig. 2C, the existence of both the unfavorable elements dramatically reduced the efficiency to an undetectable level. In addition to high GC content, we assumed that during the reaction, the three LoxP sites, one on the homologous arm and two on the two ends of the SM, disturbed the assembly process and led to the failed assembly or the formation of incomplete constructs (Supplementary Fig. S4B).

In order to examine the influence of LoxP sites, we compared the Gibson assembly efficiency of the IF with and without flanking LoxP sites. EGFP, SM (neomycin-resistance gene), and SM-EGFP were the IFs used in the experiments to also test whether the length of IFs would affect the outcome. The size of EGFP, SM, and SM-EGFP was around 0.75 kb, 1.3 kb, and 2.2 kb, respectively. As shown in the left part of Supplementary Fig. S4A, the IF vector, the 5’/3’ homologous arms from *CDH1*, and the IF were mixed to conduct Gibson assembly. Here, we would like to confirm the effect of the linker pair again. Thus, the IF vector carrying linker pair 1 and the control mentioned in Fig. 2C were used. Afterward, the colonies were verified by PCR (Supplementary Table S6 and Supplementary Fig. S5) and the quantitative results were shown in Fig. 3C. The efficiency of Gibson assembly was strongly reduced when the IF was flanked by LoxP sites. For some samples, there were even no colonies grown on the plates. On the other hand, regarding the samples without LoxP sites, the length of IF slightly influenced Gibson assembly. However, compared to LoxP sites, the effects caused by the IF with different lengths were minor, indicating that it was not the length of IF but the LoxP sites leading to the reduction of assembly efficiency. In addition, linker pair 1 significantly improved the outcome for the three IFs. Conclusively, our data proved that removable SM severely decreased the efficiency of Gibson assembly, thereby pointing out the challenges of applying the CRISPR knock-in method to silent genes, especially for the ones with unbalanced GC content around the inserted regions.

### A two-step procedure not only overcomes difficulties with unbalanced GC content and removable SM, but also allows adding sgRNA recognition sites for increasing HDR efficiency

**Figure 4:**
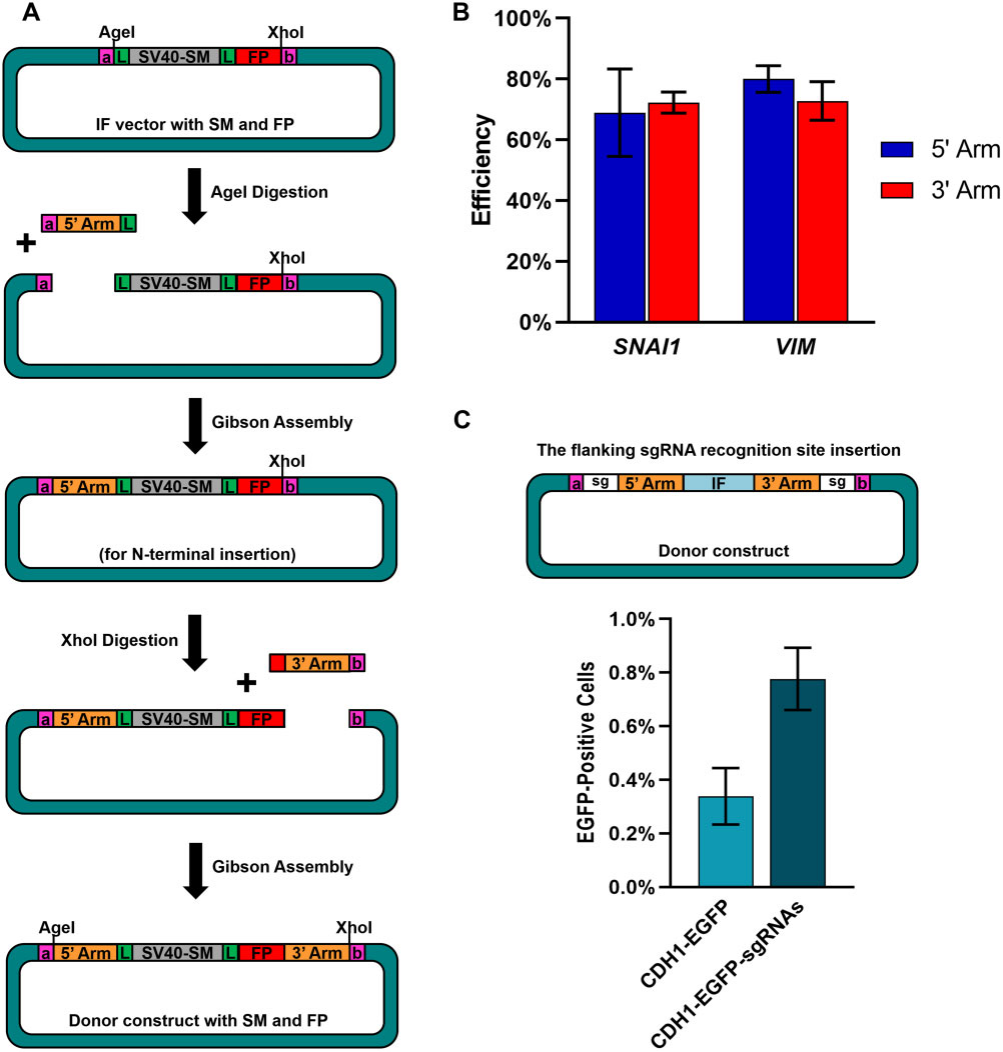
The linker pairs together with a two-step strategy facilitate the efficiency for generating the donor templates with unbalanced GC content and removable SM and allow insertion of sgRNA recognition sites to enhance the CRISPR knock-in outcome. (**A**) Schematic diagram of the cloning process with the linker pairs and the two-step design. We first generated the IF vector containing an FP as well as a removable SM driven by SV40 promoter. After two subsequent digestion and assembly procedures, the 5’ and 3’ homologous arms were integrated step-by-step to form the complete donor construct in a correct order. (**B**) The efficiency outcome of each assembly reaction for producing the donor templates targeting *SNAI1* and *VIM*. After each assembly step, we picked 60 colonies for PCR confirmation and calculated the efficiency as the number of the desirable colonies divided by the number of tested colonies. The experiments were performed in triplicate. The error bars represent mean ± SD. (**C**) Efficiency of the CRISPR knock-in experiments using the donor templates with and without sgRNA recognition sites. The sgRNA recognition sites were inserted into the donor construct targeting *CDH1* via the RESs on the linkers. The donor constructs were transfected into T47D cells to test the knock-in effects. The efficiency was defined as the number of EGFP-positive cells divided by the number of total cells. The experiments were conducted in duplicate. The error bars indicate mean ± SD. a: linker a, b: linker b, L: LoxP, SV40-SM: SV40 promoter and the SM, and sg: sgRNA recognition site.

To overcome these difficulties, we introduced a two-step Gibson assembly procedure (Fig. 4A) to increase the efficiency and to minimize the number of undesirable products from assembly reaction. The process took advantage of the RESs on the linkers as well as the hierarchical framework [23]. Again, we used *SNAI1* or *VIM* as examples to confirm our approach. First, the IF vectors carrying the linker pair 1, removable SM, and the FP were proceeded for RE, *Age*I, digestion. Next, the 5’ homologous arm was incorporated into the vectors through Gibson assembly. Then, the previous two steps were repeated but with *Xho*I digestion and 3’ homologous arm integration. To produce a donor construct with a removable SM and an FP for *SNAI1*, we successfully achieved an assembly efficiency at approximately 70% for both the 5’ and 3’ homologous arm incorporation steps (Fig. 4B;Supplementary Table S7 and Supplementary Fig. S6). Similar results were reproduced when constructing the donor template for *VIM*, a mesenchymal marker [26] that is a silent gene and has around 70% GC content in both the 5’ and 3’ homologous arms (Supplementary Table S5). Our results proved that the two-step strategy remarkably enhanced the efficiency of Gibson assembly reaction for making the donor templates containing unbalanced GC sequences and a removable SM.

In addition to the advantages mentioned above, the linkers also allowed investigators to incorporate the sgRNA recognition sites to both ends of the IFs in the donor constructs via the RESs (Fig. 4C). The sgRNA recognition sites are composed of the sgRNA target sequences as well as the PAM (NGG) sequences and have been proved to significantly increase the HDR efficiency [9]. Here, we transfected the *CDH1*-*EGFP* donor construct harboring two sgRNA recognition sites, which were inserted through the RESs on the linkers, together with deactivated Cas9 and sgRNA plasmids into T47D cells and checked the knock-in outcome by flow cytometry. The data confirmed that the *CDH1*-*EGFP-sgRNA* construct led to a two-fold higher knock-in efficiency than the donor construct without the flanking sgRNA recognition sites (Fig. 4C;Supplementary Fig. S7), which was consistent with the previous study [9]. The fluorescence images in Supplementary Fig. S8 showed the expected accumulation of EGFP at cell membrane for the successfully edited cells. In conclusion, we combined the computational selected linkers with the two-step Gibson assembly reaction to considerably enhance the accuracy and efficiency of generating the donor constructs with unbalanced GC content and removable SMs, and eventually facilitate the procedure of CRISPR knock-in approaches. 

## Discussion

The CRISPR-based gene editing techniques open the door to various fields of basic and applied science. However, as for gene knock-in, the whole process adds an additional layer of challenge since it is usually time-consuming and costly to produce the donor templates. In this research, we conducted in silico screening to select the RESs containing low identity to the human genome and used them as the linkers to ameliorate Gibson assembly reaction. Our results proved that the existence of the linkers led to up to eight-fold higher assembly rate. Meanwhile, we found that approximately 45% of human genes have unbalanced GC content at one or both homologous arm regions, which affected the Gibson assembly efficiency strongly. Moreover, including a removable SM also influenced Gibson assembly due to the flanking LoxP sites. To solve this issue, we further introduced the two-step Gibson assembly process, integrating the linkers and the hierarchical framework to improve the outcome of generating the donor templates harboring unbalanced GC content and the removable SMs, with the data revealing that the new approach caused approximately 70% assembly efficiency. Here, we want to emphasize that our method is working regardless of GC content at homologous arm regions. One-step assembly is for the sequences with regular GC content, while two-step assembly is for those with unbalanced GC content. For the latter, the one-step procedure simply fails in some difficult cases, and the slightly additional steps are necessary. Finally, the RESs on the linkers also facilitated the procedure of inserting sgRNA recognition sites to the donor constructs. We confirmed that the knock-in efficiency for the donor constructs with flanking sgRNA recognition sites, which were easily inserted through the RESs on the linkers, was two-fold higher than the ones without insertion. Taken together, the linker pairs and the two-step assembly process make the donor template generation efficient and convenient and increase the possibility for applying CRISPR editing techniques to the genes with unbalanced GC content or low expression, saving time and cost.

In principle, direct synthesis of donor constructs is an alternative strategy, but several factors limit its practical applications. First, the cost for synthesis can escalate quickly with the construct size. For example, for a donor construct containing the homologous arms, the FP and the removable SM, the size could be up to 9 kb. Another problem is that it imposes technical difficulty in synthesizing donor constructs for genes with GC-rich sequences and sequences with repeats. In comparison, the method presented in this study is cost-effective and applicable for cloning GC-rich sequences. One may also consider the RE digestion and ligation system. A main concern here is that one must carefully confirm that the RESs used for cloning are not present in vector backbone and IFs. In our design, we deliberately selected the RESs with low occurrence density in the human genome to compose the linkers. Thus, the approach can be employed to construct various genes without repeatedly checking the situation mentioned above. Moreover, the sequence-dependent ligation method results in additional RESs between the homologous arms and the IFs. It is undesirable to have extra RESs inserted into the human genome since it is unclear whether the RESs influence the function of an inserted protein. Thereby, we decided to optimize Gibson assembly to obtain “seamless” donor constructs, considering that the only two linkers we used are located outside the homologous arms and would not be inserted into the human genome.

**Figure 5:**
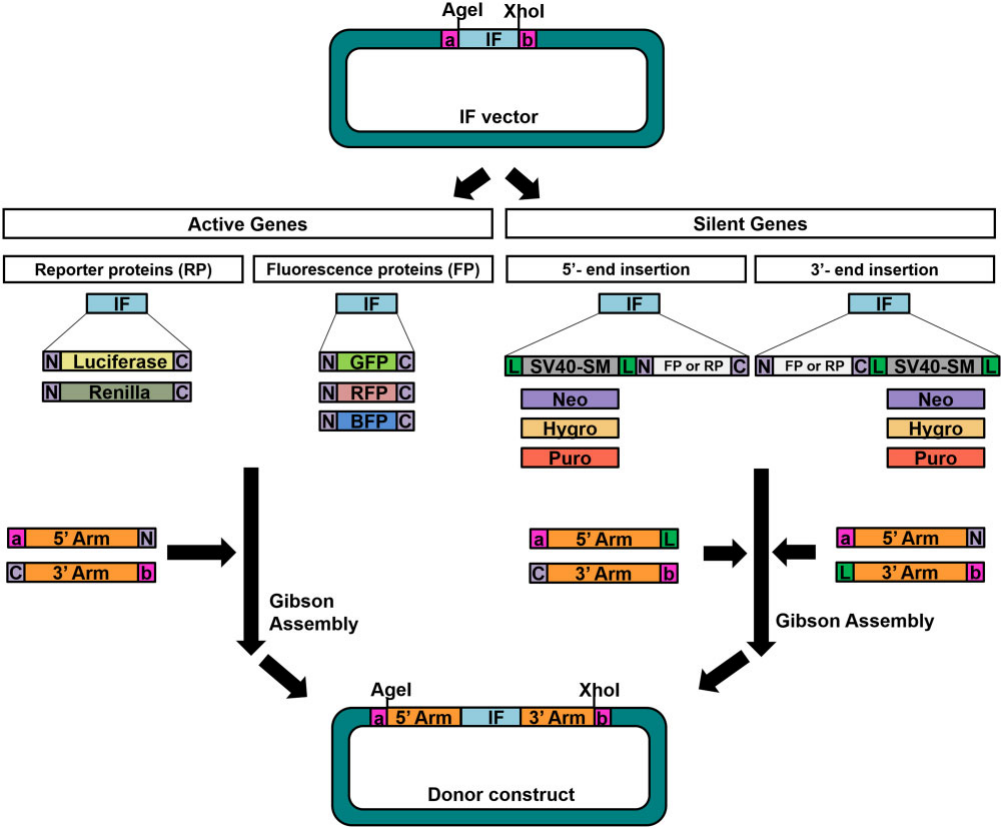
The donor vector bank provides various tools for studying different types of genes using the CRISPR knock-in technique. The IF donor vector is basically composed of a linker pair and an IF. For active genes, the IF is usually an RP or an FP. The protein linkers are optional components added based on investigators’ purposes. As for silent genes, an additional removable SM (LoxP-SM-LoxP) is required for colony selection. Thus, the IF vector has an RP/FP placed before or after a removable SM. In the donor vector bank, the options for the IF vectors are Luciferase, Renilla, GFP, RFP, or BFP. For SM, the vectors with Neomycin-, Hygromycin-, or Puromycin-resistance gene are ready. Consequently, researchers pick an IF vector and then insert the 5’/3’ homologous arm of the knock-in target genes. With the presence of the linker pair and the vector bank, donor constructs can be produced quickly and cost-effectiveness. N: protein linker N, C: protein linker C, Neo: Neomycin-resistance gene, Hygro: Hygromycin-resistance gene, Puro: Puromycin-resistance gene, L: LoxP, SV40-SM: SV40 promoter and the SM, a: linker pair a, and b: linker pair b.

Besides the benefit already stated, the method we introduced can be expanded to more applications. First, the computational pipeline can be applied to other species. For studying human genome, we already selected 12 primer pairs. Researchers can directly apply the sequences of these primer pairs to their experiments without running the program again. On the other hand, for researchers who want to generate knock-in constructs from other species, they can perform the same in silico screening, using the genome of the target species as the reference database. Second, researchers can add more fragments according to their experimental purposes through the RESs on the linkers. For example, for producing recombinant fusion proteins, it is recommended to have protein linkers between the two component proteins [31]. By using our approach, researchers can easily integrate the protein linkers at the N and/or C terminus of the inserted proteins. Third, different RPs, like Firefly and Renilla luciferases, FPs, including GFP, RFP, and BFP, as well as SMs, for example, Neomycin-, Hygromycin-, and Puromycin-resistance genes, can be simply exchanged or combined in the donor constructs (Fig. 5).

Altogether, our aim is to create a donor vector bank containing the vectors with various RPs, FPs, and/or SMs. Investigators choose the donor vectors based on the type of genes they study and then proceed to the one- or two-step assembly reaction to complete the process. For active genes that generally have a certain degree of expression, one can insert RPs or FPs for functional study. On the other hand, for silent genes that have low expression under normal cellular conditions, adding removable SMs assists the investigators to pick the desirable edited cells after performing CRISPR knock-in experiments. Ultimately, our work will improve the accessibility and popularity of CRISPR-based gene editing techniques in cell biology research, disease modeling, and synthetic biology studies. For example, one can study the functions of new genes by labeling and monitoring the temporal–spatial dynamics of the endogenous gene product [32]. One can generate models of fusion genes to investigate the physiological consequences of cancer development [33]. One can also insert regulatory elements at designated locations of a genome to manipulate the regulatory network for designed functions [34, 35]. All these experiments will be easier, faster, and cheaper by applying our method, hence, in the end, benefiting the research in the related fields.

## Author contributions

Conceptualization and Project Design: Y.-J.C. and J.X.; Project Administration and Supervision: J.X.; Software and Computational Analyses: W.W., X.-J.T., D.E.L, and J.Z.; Experiments and Data Analysis: Y.-J.C., Y.-Y.C., and D.A.T.; Writing—original draft: Y.-J.C. and J.X.; Writing—review and editing: Y.-J.C., Y.-Y.C., W.W., X.-J.T., D.E.L, D.A.T., J.Z., and J.X.

## Funding

This work was supported by the National Science Foundation [DMS-1462049 to J.X.] and National Institute of Diabetes and Digestive and Kidney Diseases (R01DK119232 to J.X.).

*Conflict of interest statement*. None declared. 

## Supplementary Material

bpaa006_Supplementary_Data
