# Erratum to: Rapid, modular, and cost-effective generation of donor DNA constructs for CRISPR-based gene knock-in

**DOI:** 10.1093/biomethods/bpaa021

**Published:** 2020-11-18

**Authors:** Yi-Jiun Chen, Ya-Yun Cheng, Weikang Wang, Xiao-Jun Tian, Daniel E Lefever, David A Taft, Jingyu Zhang, Jianhua Xing

**Affiliations:** b1 Department of Computational and Systems Biology, School of Medicine, University of Pittsburgh, Pittsburgh, PA 15260, USA; b2 Drug Discovery Institute, School of Medicine, University of Pittsburgh, Pittsburgh, PA 15260, USA

*Biology Methods and Protocols, Volume 5, Issue 1, 2020, bpaa006*. https://doi.org/10.1093/biomethods/bpaa006

The Publisher apologises for an error in the corresponding authors’ details. Jianhua Xing and Yi-Jiun Chen are both corresponding authors. The published article has been updated.

