# Supplementary material for: Rapid, modular, and cost-effective generation of donor DNA constructs for CRISPR-based gene knock-in
Source: Biol Methods Protoc. 2020 Mar 20;5(1):bpaa006. doi: 10.1093/biomethods/bpaa006 (PMC7211398; doi:10.1093/biomethods/bpaa006)
Supplement: bpaa006_Supplementary_Data [file bpaa006_supplementary_data.docx]

**SUPPLEMENTARY TABLES AND FIGURES**

**Supplementary Table 1. List of restriction enzyme sites with an occurrence density less than 300 per mega base.**

We referenced the distribution of RESs in the human genome reported on the NEB website (http://tools.neb.com/~posfai/TheoFrag/TheoreticalDigest.human.html) and listed 30 RESs with an occurrence density less than 300 per mega base. Eight RESs, marked in bold and light grey, were selected for the linker design. *: AT rich, **: the length of the RES was too long.

| **Enzyme** | **Specificity** | **Occurrence density  (per mega base)** | **Candidate** |
| --- | --- | --- | --- |
| **NotI** | GCGGCCGC | 3.3 | √ |
| MluI | ACGCGT | 7.3 |  |
| SalI | GTCGAC | 10.7 |  |
| PmeI | GTTTAAAC | 14.3 |  |
| **AgeI** | ACCGGT | 18.4 | √ |
| **ClaI** | ATCGAT | 29.7 | √ |
| **XhoI** | CTCGAG | 41.9 | √ |
| PacI | TTAATTAA | 55.4 | * |
| NheI | GCTAGC | 98.2 |  |
| SwaI | ATTTAAAT | 78.8 | * |
| KpnI | GGTACC | 100.1 |  |
| BamHI | GGATCC | 126.1 |  |
| SapI | GCTCTTC | 130.5 |  |
| SmaI | CCCGGG | 130.9 |  |
| **HpaI** | GTTAAC | 135.3 | √ |
| SpeI | ACTAGT | 137.4 |  |
| **EcoRV** | GATATC | 150.5 | √ |
| **ApaI** | GGGCCC | 159.8 | √ |
| ScaI | AGTACT | 188.5 |  |
| SphI | GCATGC | 190.7 |  |
| AvrII | CCTAGG | 206.5 |  |
| SacI | GAGCTC | 208 |  |
| NcoI | CCATGG | 262.6 |  |
| BglII | AGATCT | 269.3 |  |
| EcoRI | GAATTC | 272.1 |  |
| **XbaI** | TCTAGA | 279.3 | √ |
| StuI | AGGCCT | 279.7 |  |
| XcmI | CCANNNNNNNNNTGG | 285.3 | ** |
| HindIII | AAGCTT | 292.6 |  |

**Supplementary Table 2. Top 12 ranked linker pairs selected by in silico screening.**

Each linker pair comprised a and b. The GC content and Tm were calculated by the NEB Tm Calculator v 1.9.4 (available at http://tmcalculator.neb.com/#!/) with the Q5 High-Fidelity DNA polymerase setting. The linkers marked in bold and light grey were used for experimental testing in this study.

| **Linker pair  (rank)** | | **Length  (bp)** | **Sequence** | **GC content (%)** | **Annealing Tm (ºC)** |
| --- | --- | --- | --- | --- | --- |
| **1** | a | 30 | TCGGGCCCTTCTAGACAATCGATCACCGGT | 57 | 78 |
|  | b | 30 | CTCGAGTGTTAACGGCGGCCGGGATATCAT | 57 | 77 |
| 2 | a | 30 | TCGGGCCCTTCTAGACAATCGATCACCGGT | 57 | 78 |
|  | b | 30 | CTCGAGTGTTAACGGCGGCCGGGATATCTT | 57 | 77 |
| 3 | a | 30 | GAGGGCCCTTCTAGACAATCGATCACCGGT | 57 | 76 |
|  | b | 30 | CTCGAGTGTTAACGGCGGCCGGGATATCAT | 57 | 77 |
| 4 | a | 30 | GAGGGCCCATCTAGAACATCGATCACCGGT | 57 | 77 |
|  | b | 30 | CTCGAGTGTTAACGGCGGCCGGGATATCAT | 57 | 77 |
| 5 | a | 30 | GAGGGCCCTTCTAGACAATCGATCACCGGT | 57 | 76 |
|  | b | 30 | CTCGAGTGTTAACGGCGGCCGGGATATCTA | 57 | 76 |
| **6** | a | 30 | GTGGGCCCATCTAGAACATCGATCACCGGT | 57 | 77 |
|  | b | 30 | CTCGAGTGTTAACGGCGGCCGGGATATCTT | 57 | 77 |
| 7 | a | 30 | GAGGGCCCATCTAGACAATCGATCACCGGT | 57 | 77 |
|  | b | 30 | CTCGAGTGTTAACGGCGGCCGGGATATCTT | 57 | 77 |
| 8 | a | 30 | AAGGGCCCTTCTAGACAATCGATCACCGGT | 53 | 76 |
|  | b | 30 | CTCGAGTGTTAACGGCGGCCGGGATATCTT | 57 | 77 |
| 9 | a | 30 | AAGGGCCCTTCTAGACAATCGATCACCGGT | 53 | 76 |
|  | b | 30 | CTCGAGTGTTAACGGCGGCCGGGATATCTA | 57 | 76 |
| 10 | a | 30 | CAGGGCCCTTCTAGACAATCGATCACCGGT | 57 | 77 |
|  | b | 30 | CTCGAGTGTTAACGGCGGCCGGGATATCAT | 57 | 77 |
| 11 | a | 30 | CAGGGCCCTTCTAGACAATCGATCACCGGT | 57 | 77 |
|  | b | 30 | CTCGAGTGTTAACGGCGGCCGGGATATCTT | 57 | 77 |
| 12 | a | 30 | CAGGGCCCTTCTAGACAATCGATCACCGGT | 57 | 77 |
|  | b | 30 | CTCGAGTGTTAACGGCGGCCGGGATATCTA | 57 | 76 |
| **MODAL (positive control)** | a | 30 | CACTCTGGGACTGAAACTTCGTTCGGCGAC | 57 | 76 |
|  | b | 30 | CCACGGTCAGTGTCACCTGGCGGCGTCTGT | 67 | 83 |
| **Vector sequence  (negative control)** | a | 30 | AGATATCCAGCACAGTGGCGGCCGCTCGAG | 63 | 81 |
|  | b | 30 | TCTAGAGGGCCCGTTTAAACCCGCTGATCA | 53 | 76 |

**Supplementary Table 3. PCR primers for amplifying the 5’/3’ homologous arm of the knock-in target genes.**

For one-step Gibson assembly, the primers consisted of two parts: (i) 30 bps of the homologous sequences from the knock-in target genes (purple) and (ii) 30 bps of the linker sequences (black) or the FP sequences (light green). While for two-step Gibson assembly, the primers were composed of (i) 30 bps of the homologous sequences from the knock-in target genes (purple) and (ii) 30 bps of the linker sequences (black), the FP sequences (red), or the SM sequences (dark green). All the Tms were calculated by the NEB Tm Calculator mentioned in Supplemental Table 1.

| **Primer name** | | **Primer Sequence (5'→3')** | **Linker (5')  GC content (%)** | **Linker (5')  Tm (ºC)** | **Annealing (3')  GC content (%)** | **Annealing (3')  Tm (ºC)** |
| --- | --- | --- | --- | --- | --- | --- |
| **For one-step Gibson** | |  |  |  |  |  |
| *CDH1* | 5'arm-control-a-F | **AGATATCCAGCACAGTGGCGGCCGCTCGAG**ACAAGTCCTTCCTTAGTTCCCACACCCCCA | 63 | 81 | 53 | 65 |
|  | 3'arm-control-b-R | **TCTAGAGGGCCCGTTTAAACCCGCTGATCA**GGGTAACACAGGGAGACCCCGTCTCAAATA | 53 | 76 | 53 | 75 |
|  | 5'arm-1a-F | **TCGGGCCCTTCTAGACAATCGATCACCGGT**ACAAGTCCTTCCTTAGTTCCCACACCCCCA | 57 | 78 | 53 | 65 |
|  | 3'arm-1b-R | **ATGATATCCCGGCCGCCGTTAACACTCGAG**GGGTAACACAGGGAGACCCCGTCTCAAATA | 57 | 77 | 53 | 75 |
|  | 5'arm-6a-F | **GTGGGCCCATCTAGAACATCGATCACCGGT**ACAAGTCCTTCCTTAGTTCCCACACCCCCA | 57 | 77 | 53 | 65 |
|  | 3'arm-6b-R | **AAGATATCCCGGCCGCCGTTAACACTCGAG**GGGTAACACAGGGAGACCCCGTCTCAAATA | 57 | 77 | 53 | 75 |
|  | MODAL-5'arm-a-F | **CACTCTGGGACTGAAACTTCGTTCGGCGAC**ACAAGTCCTTCCTTAGTTCCCACACCCCCA | 57 | 76 | 53 | 65 |
|  | MODAL-3'arm-b-R | **CCACGGTCAGTGTCACCTGGCGGCGTCTGT**GGGTAACACAGGGAGACCCCGTCTCAAATA | 67 | 83 | 53 | 75 |
|  | 5'arm-FP-R | **GGTGAACAGCTCCTCGCCCTTGCTCACGAT**GTCGTCCTCGCCGCCTCCGTACATGTCAGC | 60 | 79 | 67 | 81 |
|  | 3'arm-FP-F | **ACTCTCGGCATGGACGAGCTGTACAAGTAA**TAGGGGACTCGAGAGAGGCGGGCCCCAGAC | 50 | 74 | 70 | 83 |
| *CTNNB1* | 5'arm-control-a-F | **AGATATCCAGCACAGTGGCGGCCGCTCGAG**GTATCCATATTGGTAATATTTGTATTTATA | 63 | 81 | 20 | 56 |
|  | 3'arm-control-b-R | **AGGTGACACTATAGAATAGGGCCCTCTAGA**AAACTATTTTACACTAACTTTTTAGTTCTC | 53 | 76 | 23 | 59 |
|  | 5'arm-1a-F | **TCGGGCCCTTCTAGACAATCGATCACCGGT**GTATCCATATTGGTAATATTTGTATTTATA | 57 | 78 | 20 | 56 |
|  | 3'arm-1b-R | **ATGATATCCCGGCCGCCGTTAACACTCGAG**AAACTATTTTACACTAACTTTTTAGTTCTC | 57 | 77 | 23 | 59 |
|  | 5'arm-6a-F | **GTGGGCCCATCTAGAACATCGATCACCGGT**GTATCCATATTGGTAATATTTGTATTTATA | 57 | 77 | 20 | 56 |
|  | 3'arm-6b-R | **AAGATATCCCGGCCGCCGTTAACACTCGAG**AAACTATTTTACACTAACTTTTTAGTTCTC | 57 | 77 | 23 | 59 |
|  | MODAL-5'arm-a-F | **CACTCTGGGACTGAAACTTCGTTCGGCGAC**GTATCCATATTGGTAATATTTGTATTTATA | 57 | 76 | 20 | 56 |
|  | MODAL-3'arm-b-R | **CCACGGTCAGTGTCACCTGGCGGCGTCTGT**AAACTATTTTACACTAACTTTTTAGTTCTC | 67 | 83 | 23 | 59 |
|  | 5'arm-FP-R | **GGTGAACAGCTCCTCGCCCTTGCTCACGAT**TGTCCACGCTGGATTTTCAAAACAGTTGTA | 60 | 79 | 40 | 70 |
|  | 3'arm-FP-F | **ACTCTCGGCATGGACGAGCTGTACAAGTAA**ATCGCTACTCAAGGTTTGTGTCATTAAATC | 50 | 74 | 37 | 67 |
| *SNAI1* | 5'arm-control-a-F | **AGATATCCAGCACAGTGGCGGCCGCTCGAG**AGCCACGTGCGGTGTCCCTTTCCTCGCTTC | 63 | 81 | 63 | 81 |
|  | 3'arm-control-b-R | **AGGTGACACTATAGAATAGGGCCCTCTAGA**GTCGTAGGGCTGCTGGAAGGTAAACTCTGA | 53 | 76 | 53 | 75 |
|  | 5'arm-1a-F | **TCGGGCCCTTCTAGACAATCGATCACCGGT**AGCCACGTGCGGTGTCCCTTTCCTCGCTTC | 57 | 78 | 63 | 81 |
|  | 3'arm-1b-R | **ATGATATCCCGGCCGCCGTTAACACTCGAG**GTCGTAGGGCTGCTGGAAGGTAAACTCTGA | 57 | 77 | 53 | 75 |
|  | 5'arm-6a-F | **GTGGGCCCATCTAGAACATCGATCACCGGT**AGCCACGTGCGGTGTCCCTTTCCTCGCTTC | 57 | 77 | 63 | 81 |
|  | 3'arm-6b-R | **AAGATATCCCGGCCGCCGTTAACACTCGAG**GTCGTAGGGCTGCTGGAAGGTAAACTCTGA | 57 | 77 | 53 | 75 |
|  | MODAL-5'arm-a-F | **CACTCTGGGACTGAAACTTCGTTCGGCGAC**AGCCACGTGCGGTGTCCCTTTCCTCGCTTC | 57 | 76 | 63 | 81 |
|  | MODAL-3'arm-b-R | **CCACGGTCAGTGTCACCTGGCGGCGTCTGT**GTCGTAGGGCTGCTGGAAGGTAAACTCTGA | 67 | 83 | 53 | 75 |
|  | 5'arm-FP-R | **GGTGAACAGCTCCTCGCCCTTGCTCACGAT**AGTGGTCGAGGCACTGGGGTCGCCGATTCG | 60 | 79 | 67 | 83 |
|  | 3'arm-FP-F | **ACTCTCGGCATGGACGAGCTGTACAAGTAA**ATCCCGCGCTCTTTCCTCGTCAGGAAGCCC | 50 | 74 | 63 | 81 |
| **For two-step Gibson** | |  |  |  |  |  |
| *SNAI1* | 5'arm-1a-F | **TCGGGCCCTTCTAGACAATCGATCACCGGT**AGCCACGTGCGGTGTCCCTTTCCTCGCTTC | 57 | 78 | 63 | 81 |
|  | 5'arm-LoxP-R | **ATAACTTCGTATAGCATACATTATACGAAG**AGTGGTCGAGGCACTGGGGTCGCCGATTCG | 30 | 62 | 67 | 83 |
|  | 3'arm-FP-F | **CTTCGTATAGCATACATTATACGAAGTTAT**ATCCCGCGCTCTTTCCTCGTCAGGAAGCCC | 30 | 62 | 63 | 81 |
|  | 3'arm-1b-R | **ATGATATCCCGGCCGCCGTTAACACTCGAG**GTCGTAGGGCTGCTGGAAGGTAAACTCTGA | 57 | 77 | 53 | 75 |
| *VIM* | 5'arm-1a-F | **TCGGGCCCTTCTAGACAATCGATCACCGGT**AGGCAAGTCGATGGACAGAGGCGCGGGCCG | 57 | 78 | 70 | 85 |
|  | 5'arm-LoxP-R | **ATAACTTCGTATAGCATACATTATACGAAG**GGCTGCGGAGGGTGGCGATGGCCTGGGCGG | 30 | 62 | 80 | 90 |
|  | 3'arm-FP-F | **CTTCGTATAGCATACATTATACGAAGTTAT**ATCTCCACCAGGTCCGTGTCCTCGTCCTCC | 30 | 62 | 63 | 81 |
|  | 3'arm-1b-R | **ATGATATCCCGGCCGCCGTTAACACTCGAG**TGCAAGGTCTGGGTTCTGGGCGGGGCTGCG | 57 | 77 | 70 | 85 |

**Supplementary Table 4. The number of colonies obtained after Gibson assembly for studying the effect of linker pairs.**

After performing Gibson assembly, the colonies on the plates were counted and verified by PCR. The efficiency was calculated by dividing the number of correct colonies by the number of tested colonies. *: for the samples with more than 100 colonies, only 60 colonies were checked. N/A: not available from the samples, of which the homologous arms could not be amplified by PCR.

| **Gene** | **Sample** | **Replicate** | **Tested colonies** | **Correct colonies** | **Efficiency (%)** |
| --- | --- | --- | --- | --- | --- |
| *CDH1* | Control | 1 | 60* | 5 | 8.33 |
|  |  | 2 | 60* | 2 | 3.33 |
|  |  | 3 | 51 | 5 | 9.80 |
|  | Pair 1 | 1 | 14 | 8 | 57.14 |
|  |  | 2 | 5 | 4 | 80.00 |
|  |  | 3 | 12 | 9 | 75.00 |
|  | Pair 6 | 1 | 23 | 5 | 21.74 |
|  |  | 2 | 60* | 6 | 10.00 |
|  |  | 3 | 30 | 8 | 26.67 |
|  | MODAL | 1 | 60* | 21 | 35.00 |
|  |  | 2 | 60* | 27 | 45.00 |
|  |  | 3 | 60* | 32 | 53.33 |
| *CTNNB1* | Control | 1 | 60* | 4 | 6.67 |
|  |  | 2 | 60* | 7 | 11.67 |
|  |  | 3 | 60* | 6 | 10.00 |
|  | Pair 1 | 1 | 18 | 6 | 33.33 |
|  |  | 2 | 15 | 8 | 53.33 |
|  |  | 3 | 12 | 9 | 75.00 |
|  | Pair 6 | 1 | 37 | 6 | 16.22 |
|  |  | 2 | 33 | 10 | 30.30 |
|  |  | 3 | 28 | 9 | 32.14 |
|  | MODAL | 1 | N/A | N/A | N/A |
|  |  | 2 | N/A | N/A | N/A |
|  |  | 3 | N/A | N/A | N/A |
| *SNAI1* | Control | 1 | 14 | 1 | 7.14 |
|  |  | 2 | 47 | 7 | 14.89 |
|  |  | 3 | 25 | 3 | 12.00 |
|  | Pair 1 | 1 | 4 | 1 | 25.00 |
|  |  | 2 | 48 | 13 | 27.08 |
|  |  | 3 | 10 | 1 | 10.00 |
|  | Pair 6 | 1 | 5 | 1 | 20.00 |
|  |  | 2 | 72 | 28 | 38.89 |
|  |  | 3 | 13 | 3 | 23.08 |
|  | MODAL | 1 | 20 | 2 | 10.00 |
|  |  | 2 | 12 | 0 | 0.00 |
|  |  | 3 | 32 | 2 | 6.25 |

**Supplementary Table 5. Sequences and GC content of the 5’/3’ homologous arm for *CDH1*, *CTNNB1*, *SNAI1*, and *VIM*.**

The DNA sequences were obtained from the NCBI website (<https://www.ncbi.nlm.nih.gov/>). The Reference Sequences of *CDH1*, *CTNNB1*, *SNAI1*, and *VIM* were NG_008021.1, NG_013302.2, NC_000020.11, and NG_012413.1, respectively. The GC contents of all fragments were calculated by the NEB Tm Calculator mentioned in Supplemental Table 1.

| **Gene** | | **Length  (bp)** | **GC content  (%)** | **Sequence** |
| --- | --- | --- | --- | --- |
| *CDH1* (C-terminal) | 5' Arm | 650 | 50 | ACAAGTCCTTCCTTAGTTCCCACACCCCCACTCCCCAGATTTAACTGTTAAGCATTTGGCATTTATCCTTCCAGGCCTTTCTGGGTGGAAATGCAGCCTGCATGCACCAGTATGCAAAGCTTCCAGCTCTGCCCATCCCAGCTGGTGGTGTGGCAGCCCCACTCTGATCTATGGGGACTCCATCCTGACTGGTTGGTGCTTGCATCTCTCCTGCCTACATATAACTCACCATTTCACTTTCTCTTTCCATCAACAACATATCCTGTGCTTCTGCATCCTTCCATGACAGTGTGTATAAATGTACGTTGTTGGTGTTCACTGCTCCGTGGTGTGCCACAAGTCTGGGTGCATTGTCGTACCTTACATATTGCTAGACTTCTTGCCCCAGATGACAGGTGTGCCCTTCCTTTCACTAAAAGATGCTTTTGTCCCTTCTTCTTTAGAATCTGAAAGCGGCTGATACTGACCCCACAGCCCCGCCTTATGATTCTCTGCTCGTGTTTGACTATGAAGGAAGCGGTTCCGAAGCTGCTAGTCTGAGCTCCCTGAACTCCTCAGAGTCAGACAAAGACCAGGACTATGACTACTTGAACGAATGGGGCAATCGCTTCAAGAAGCTGGCTGACATGTACGGAGGCGGCGAGGACGAC |
|  | 3' Arm | 666 | 42.2 | TAGGGGACTCGAGAGAGGCGGGCCCCAGACCCATGTGCTGGGAAATGCAGAAATCACGTTGCTGGTGGTTTTTCAGCTCCCTTCCCTTGAGATGAGTTTCTGGGGAAAAAAAAGAGACTGGTTAGTGATGCAGTTAGTATAGCTTTATACTCTCTCCACTTTATAGCTCTAATAAGTTTGTGTTAGAAAAGTTTCGACTTATTTCTTAAAGCTTTTTTTTTTTTCCCATCACTCTTTACATGGTGGTGATGTCCAAAAGATACCCAAATTTTAATATTCCAGAAGAACAACTTTAGCATCAGAAGGTTCACCCAGCACCTTGCAGATTTTCTTAAGGAATTTTGTCTCACTTTTAAAAAGAAGGGGAGAAGTCAGCTACTCTAGTTCTGTTGTTTTGTGTATATAATTTTTTAAAAAAAATTTGTGTGCTTCTGCTCATTACTACACTGGTGTGTCCCTCTGCCTTTTTTTTTTTTTTAAGACAGGGTCTCATTCTATCGGCCAGGCTGGAGTGCAGTGGTGCAATCACAGCTCACTGCAGCCTTGTCCTCCCAGGCTCAAGCTATCCTTGCACCTCAGCCTCCCAAGTAGCTGGGACCACAGGCATGCACCACTACGCATGACTAATTTTTTAAATATTTGAGACGGGGTCTCCCTGTGTTACCC |
| *CTNNB1*  (N-terminal) | 5' Arm | 711 | 32.1 | GTATCCATATTGGTAATATTTGTATTTATAATAAATCATTGCTGTAAATTTGAACTTAGAAAAATTTTACTAATAAAGGTGCTTTTGTGTTGCAAACTTTCATTTGAAAAGTAATTTTTCTTTGTACCAAAAAATCTAAAATTCGCTATTCTAGTCACCAAAATTTGCTTTATGAAAAATAATTTTTGATGGCACTATATCAGAAAACAACTTGTTAAAGAAAATGTGGAGTTTTTAAAATCCCACTGTACCTCTGTTATCCAAAGGGGATCTGTGAATTTTTCTGTGAAAGGTTAAAAAAGGAGAGACCTTTAGGAATTCAGAGAGCAGCTGATTTTTGAATAGTGTTTTCCCCTCCCTGGCTTTTATTATTACAACTCTGTGCTTTTTCATCACCATCCTGAATATCTATAATTAATATTTATACTATTAATAAAAAGACATTTTTGGTAAGGAGGAGTTTTCACTGAAGTTCAGCAGTGATGGAGCTGTGGTTGAGGTGTCTGGAGGAGACCATGAGGTCTGCGTTTCACTAACCTGGTAAAAGAGGATATGGGTTTTTTTTGTGGGTGTAATAGTGACATTTAACAGGTATCCCAGTGACTTAGGAGTATTAATCAAGCTAAATTTAAATCCTAATGACTTTTGATTAACTTTTTTTAGGGTATTTGAAGTATACCATACAACTGTTTTGAAAATCCAGCGTGGACA |
|  | 3' Arm | 764 | 42 | ATGGCTACTCAAGGTTTGTGTCATTAAATCTTTAGTTACTGAATTGGGGCTCTGCTTCGTTGCCATTAAGCCAGTCTGGCTGAGATCCCCCTGCTTTCCTCTCTCCCTGCTTACTTGTCAGGCTACCTTTTGCTCCATTTTCTGCTCACTCCTCCTAATGGCTTGGTGAAATAGCAAACAAGCCACCAGCAGGAATCTAGTCTGGATGACTGCTTCTGGAGCCTGGATGCAGTACCATTCTTCCACTGATTCAGTGAGTAACTGTTAGGTGGTTCCCTAAGGGATTAGGTATTTCATCACTGAGCTAACCCTGGCTATCATTCTGCTTTTCTTGGCTGTCTTTCAGATTTGACTTTATTTCTAAAAATATTTCAATGGGTCATATCACAGATTCTTTTTTTTTAAATTAAAGTAACATTTCCAATCTACTAATGCTAATACTGTTTCGTATTTATAGCTGATTTGATGGAGTTGGACATGGCCATGGAACCAGACAGAAAAGCGGCTGTTAGTCACTGGCAGCAACAGTCTTACCTGGACTCTGGAATCCATTCTGGTGCCACTACCACAGCTCCTTCTCTGAGTGGTAAAGGCAATCCTGAGGAAGAGGATGTGGATACCTCCCAAGTCCTGTATGAGTGGGAACAGGGATTTTCTCAGTCCTTCACTCAAGAACAAGTAGCTGGTAAGAGTATTATTTTTCATTGCCTTACTGAAAGTCAGAATGCAGTTTTGAGAACTAAAAAGTTAGTGTATAATAGTTT |
| *SNAI1* (N-terminal) | 5' Arm | 614 | 72.8 | AGCCACGTGCGGTGTCCCTTTCCTCGCTTCCTCCCCAGTGATGTGCGTTTCCCTCGTCAATGCCACGCTCTCCAGGCGCCAGCCGGGCGGAGGAAATTTCCGCCCCCTCCCAAGCCCGAGGCGGGGGCGGGCGTCGGAAGGTCAGGTGTCCCGGCCGGCGCGCAGCGCCAGGGGGCGTCAGAAGCGCTCAGACCACCGGGCGCTGAGCCGGTGGGCGCGCGGCGTCCTGCCGGGGTCCCACCTCGCAGAGGCCTCGCTTCGCTCGACGTCCCGCCCCGGACAGCCCCAGCACCGGGGACGACCCGCGCTGCGCCAGCGAACCCCGCCTCGGAGGAGTCCCCGCCCGGGCTCTCACCGCCACGCGGCGCGAGCCCGGCCAGCAGCCGGCGCACCTGCTCGGGGAGTGGCCTTCGGCGGAGACGAGCCTCCGATTGGCGCGGAGGTGACAAAGGGGCGTGGCAGATAAGGCCCCGCCCCTCCCACCCCCCACCACCCCCCGGAGTACTTAAGGGAGTTGGCGGCGCTGCTGCATTCATTGCGCCGCGGCACGGCCTAGCGAGTGGTTCTTCTGCGCTACTGCTGCGCGAATCGGCGACCCCAGTGCCTCGACCACT |
|  | 3' Arm | 790 | 60.5 | ATGCCGCGCTCTTTCCTCGTCAGGAAGCCCTCCGACCCCAATCGGAAGCCTAACTACAGCGAGCTGCAGGACTCTAATCCAGGTGCGTTGGAGGGGTTCTGGGCTCCAGGAGGTTTGGGGGAGACAGGCGAAGGCTGCGTGGGGGGCACCTGAGGGAGGCGGCCTGCCTGAGCCAGGATCGAGTCACAGGATGTTTTGTGGACCATTGCGGGCTCGGGAGACCGGGCAAGTGGGTCCCCAGTTCCGGGGATCTGTCTGGGTGGTTGGGGGAGTGCCGTGTAGAGGGCAGGGGTCTTCAGCTTGGGGGGCCTTTGTAGCCGGCGAGAGGCGGAGGAGCTCCGCAAGAGGGGAAGGAGAGGAGGCCTGTGTCAGGAGGGCCCTCTGGACGCTGCTGGGGAGAGTCCGGAGTCCAGAGGGTTGAGGGGAGGGGTGGGGAGACGAGATGTGTGTGAGGAGGGGGATTGGGGCAGGGTGGTGGCTCCGGGGCTGGGATGATGGGGTTCTGGCCTCAGGCTGGAGACTGGGGACTTAGGAGAGGGAGATCAGGAAATGACCTCCTTCAACTGGGGGTCCTACGTGTGAGAGACTCAGATTGGGTGACCTGGGCGAGGAGGGCAGGAACCTGGTCTGTCCTGTGGATAATTTTTTTGATCTAATTATGTATTGAGAATCGGCCCCACCCAGCCCCTGGCCAGCGGTGGGCTCATGTTTGTTGATTGAGTGAATGATTTAATTAACGCCTGACTCTGCTTTTTCTCCCTCAGAGTTTACCTTCCAGCAGCCCTACGAC |
| *VIM* (N-terminal) | 5' Arm | 748 | 69.8 | AGGCAAGTCGATGGACAGAGGCGCGGGCCGGAGCAGCCCCCCTTTCCAAGCGGGCGGCGCGCGAGGCTGCGGCGAGGCCTGAGCCCTGCGTTCCTGCGCTGTGCGCGCCCCCACCCCGCGTTCCAATCTCAGGCGCTCTTTGTTTCTTTCTCCGCGACTTCAGATCTGAGGGATTCCTTACTCTTTCCTCTTCCCGCTCCTTTGCCCGCGGGTCTCCCCGCCTGACCGCAGCCCCGAGACCGCCGCGCACCTCCTCCCACGCCCCTTTGGCGTGGTGCCACCGGACCCCTCTGGTTCAGTCCCAGGCGGACCCCCCCCTCACCGCGCGACCCCGCCTTTTTCAGCACCCCAGGGTGAGCCCAGCTCAGACTATCATCCGGAAAGCCCCCAAAAGTCCCAGCCCAGCGCTGAAGTAACGGGACCATGCCCAGTCCCAGGCCCCGGAGCAGGAAGGCTCGAGGGCGCCCCCACCCCACCCGCCCACCCTCCCCGCTTCTCGCTAGGTCCCTATTGGCTGGCGCGCTCCGCGGCTGGGATGGCAGTGGGAGGGGACCCTCTTTCCTAACGGGGTTATAAAAACAGCGCCCTCGGCGGGGTCCAGTCCTCTGCCACTCTCGCTCCGAGGTCCCCGCGCCAGAGACGCAGCCGCGCTCCCACCACCCACACCCACCGCGCCCTCGTTCGCCTCTTCTCCGGGAGCCAGTCCGCGCCACCGCCGCCGCCCAGGCCATCGCCACCCTCCGCAGCC |
|  | 3' Arm | 752 | 68.8 | ATGTCCACCAGGTCCGTGTCCTCGTCCTCCTACCGCAGGATGTTCGGCGGCCCGGGCACCGCGAGCCGGCCGAGCTCCAGCCGGAGCTACGTGACTACGTCCACCCGCACCTACAGCCTGGGCAGCGCGCTGCGCCCCAGCACCAGCCGCAGCCTCTACGCCTCGTCCCCGGGCGGCGTGTATGCCACGCGCTCCTCTGCCGTGCGCCTGCGGAGCAGCGTGCCCGGGGTGCGGCTCCTGCAGGACTCGGTGGACTTCTCGCTGGCCGACGCCATCAACACCGAGTTCAAGAACACCCGCACCAACGAGAAGGTGGAGCTGCAGGAGCTGAATGACCGCTTCGCCAACTACATCGACAAGGTGCGCTTCCTGGAGCAGCAGAATAAGATCCTGCTGGCCGAGCTCGAGCAGCTCAAGGGCCAAGGCAAGTCGCGCCTGGGGGACCTCTACGAGGAGGAGATGCGGGAGCTGCGCCGGCAGGTGGACCAGCTAACCAACGACAAAGCCCGCGTCGAGGTGGAGCGCGACAACCTGGCCGAGGACATCATGCGCCTCCGGGAGAAGTAAGGCTGCGCCCATGCAAGTAGCTGGGCCTCGGGAGGGGGCTGGAGGGAGAGGGGAACGCCCCCCCGGCCCCCGCGAGAGCTGCCACGCCCTTGGGGATGTGGCCGGGGGGAGGCCTGCCAGGGAGACAGCGGAGAGCGGGGCTGTGGCTGTGGTGGCGCAGCCCCGCCCAGAACCCAGACCTTGCA |

**Supplementary Table 6. The number of colonies obtained after Gibson assembly for evaluating the effect of LoxP sites.**

After performing Gibson assembly, the colonies on the plates were counted and verified by PCR. The efficiency was calculated by dividing the number of correct colonies by the number of tested colonies. *: for the samples with more than 100 colonies, only 60 colonies were checked. N/A: not available from the samples without any colony grown on the plates.

| **IF** | **Linker** | **LoxP site** | **Replicate** | **Tested colonies** | **Correct colonies** | **Efficiency (%)** |
| --- | --- | --- | --- | --- | --- | --- |
| EGFP | Control | - | 1 | 60* | 2 | 3.33 |
|  |  |  | 2 | 60* | 2 | 3.33 |
|  |  |  | 3 | 60* | 1 | 1.67 |
|  |  | + | 1 | 0 | N/A | N/A |
|  |  |  | 2 | 0 | N/A | N/A |
|  |  |  | 3 | 2 | 0 | 0.00 |
|  | Pair 1 | - | 1 | 60* | 33 | 55.00 |
|  |  |  | 2 | 60* | 27 | 45.00 |
|  |  |  | 3 | 59 | 29 | 49.15 |
|  |  | + | 1 | 0 | N/A | N/A |
|  |  |  | 2 | 2 | 0 | 0.00 |
|  |  |  | 3 | 3 | 0 | 0.00 |
| SM | Control | - | 1 | 60* | 7 | 11.67 |
|  |  |  | 2 | 53 | 4 | 7.55 |
|  |  |  | 3 | 23 | 1 | 4.35 |
|  |  | + | 1 | 69 | 0 | 0.00 |
|  |  |  | 2 | 0 | N/A | N/A |
|  |  |  | 3 | 0 | N/A | N/A |
|  | Pair 1 | - | 1 | 60* | 35 | 58.33 |
|  |  |  | 2 | 10 | 9 | 90.00 |
|  |  |  | 3 | 25 | 14 | 56.00 |
|  |  | + | 1 | 37 | 0 | 0.00 |
|  |  |  | 2 | 0 | N/A | N/A |
|  |  |  | 3 | 0 | N/A | N/A |
| SM-EGFP | Control | - | 1 | 60* | 6 | 10.00 |
|  |  |  | 2 | 60* | 7 | 11.67 |
|  |  |  | 3 | 60* | 6 | 10.00 |
|  |  | + | 1 | 11 | 0 | 0.00 |
|  |  |  | 2 | 63 | 0 | 0.00 |
|  |  |  | 3 | 9 | 0 | 0.00 |
|  | Pair 1 | - | 1 | 60* | 39 | 65.00 |
|  |  |  | 2 | 60* | 44 | 73.33 |
|  |  |  | 3 | 60* | 43 | 71.67 |
|  |  | + | 1 | 13 | 0 | 0.00 |
|  |  |  | 2 | 37 | 0 | 0.00 |
|  |  |  | 3 | 13 | 0 | 0.00 |

**Supplementary Table 7. The colony number obtained after Gibson assembly for testing the effect of two-step approach.**

After performing Gibson assembly, the colonies on the plates were counted and verified by PCR. The efficiency was calculated by dividing the number of correct colonies by the number of tested colonies. *: for the samples with more than 100 colonies, only 60 colonies were checked.

| **Gene** | **Sample** | **Replicate** | **Tested colonies** | **Correct colonies** | **Efficiency (%)** |
| --- | --- | --- | --- | --- | --- |
| *SNAI1* | 5' Arm | 1 | 60* | 32 | 53.33 |
|  |  | 2 | 60* | 43 | 71.67 |
|  |  | 3 | 60* | 49 | 81.67 |
|  | 3' Arm | 1 | 60* | 45 | 75.00 |
|  |  | 2 | 60* | 41 | 68.33 |
|  |  | 3 | 60* | 44 | 73.33 |
| *VIM* | 5' Arm | 1 | 60* | 46 | 76.67 |
|  |  | 2 | 60* | 51 | 85.00 |
|  |  | 3 | 60* | 47 | 78.33 |
|  | 3' Arm | 1 | 60* | 41 | 68.33 |
|  |  | 2 | 60* | 48 | 80.00 |
|  |  | 3 | 60* | 42 | 70.00 |


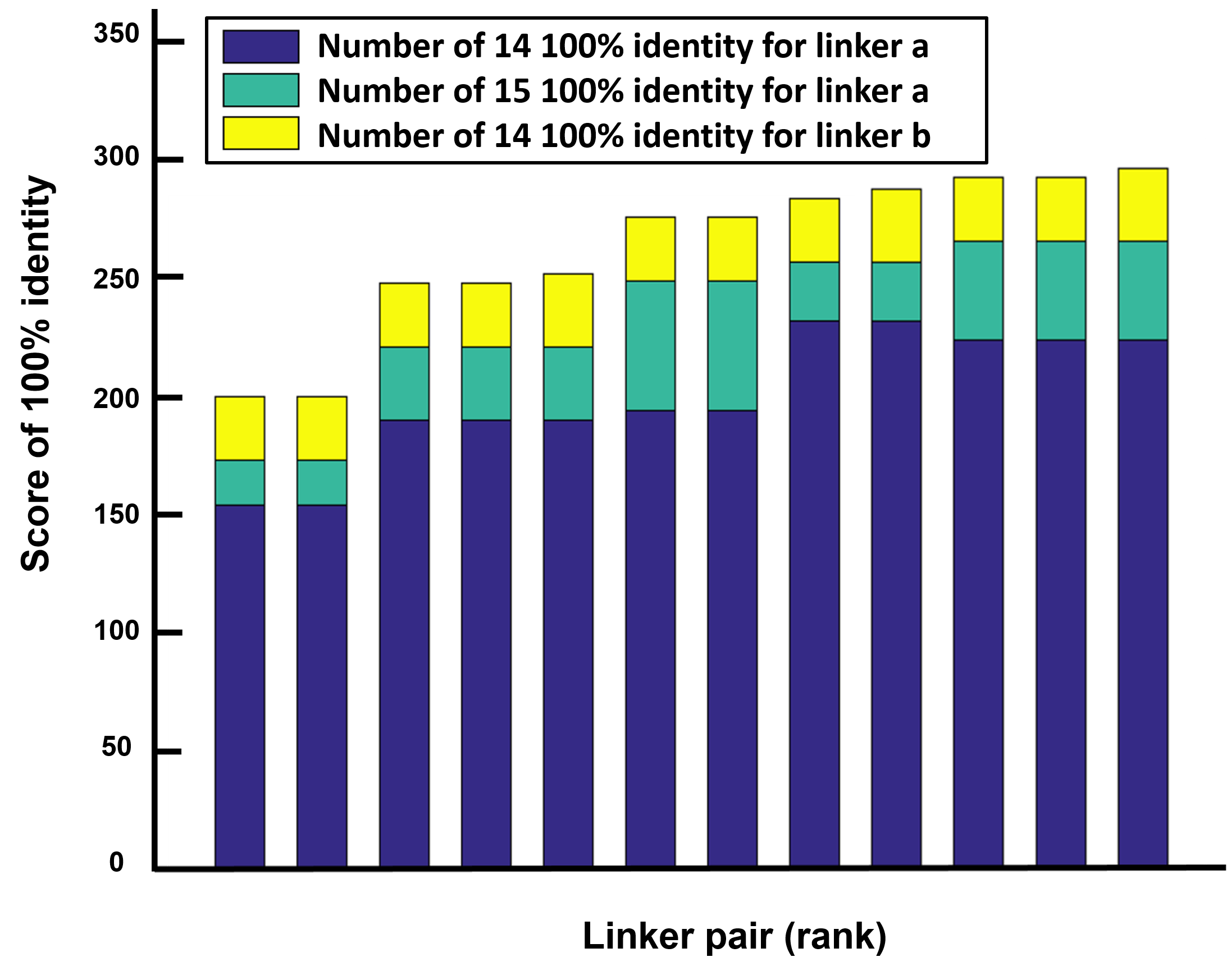


**Supplementary Figure 1.** **BLAST alignment results for the top-ranked linkers.**

The candidate linker pairs (linker a and b) were subjected to BLAST alignment procedure. During this step, the sequences of the linker pairs were compared with the human genome for checking the identity. The linker pair with the lowest identity score was ranked 1 and the results for top 12 linker pairs were shown here.


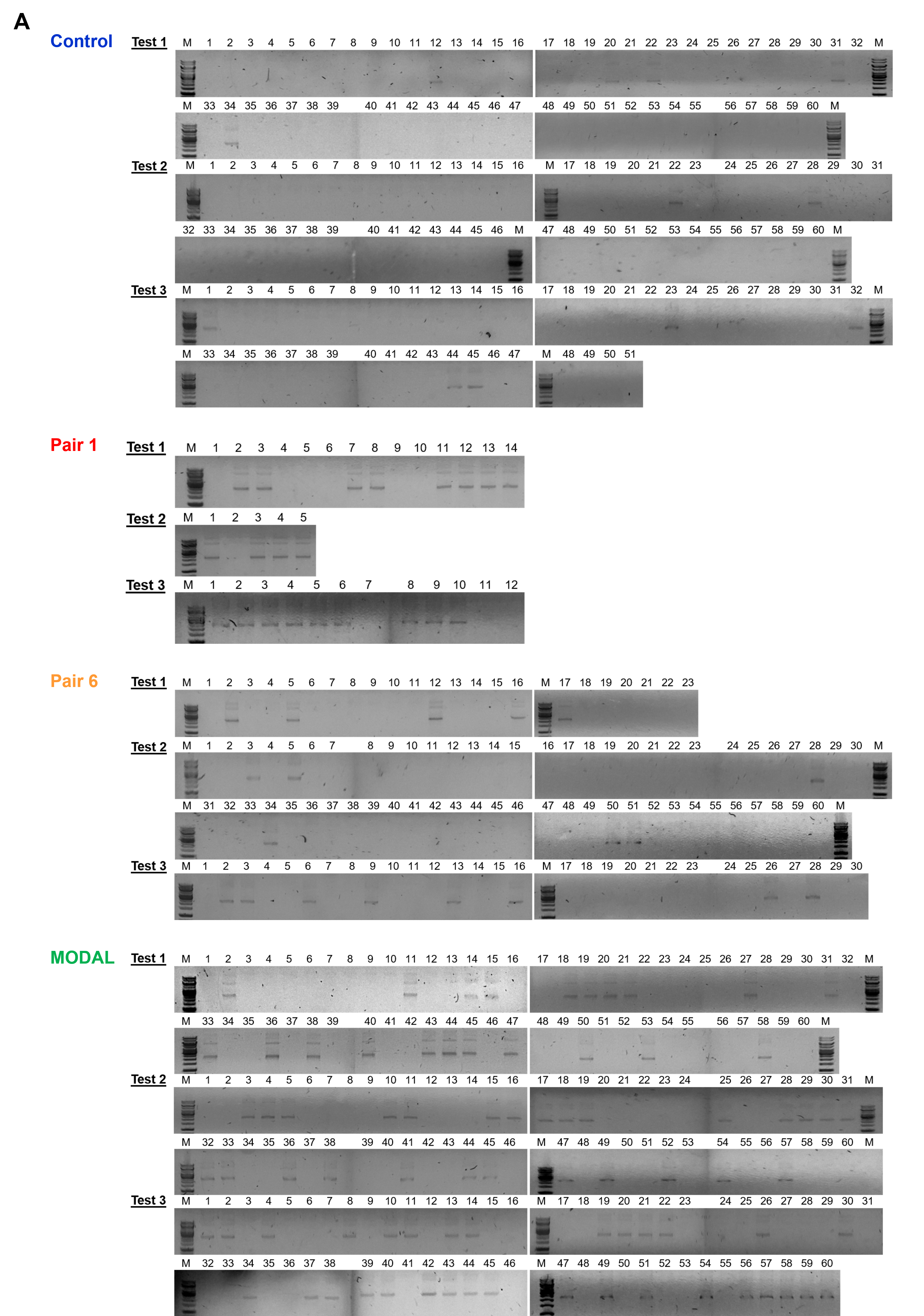


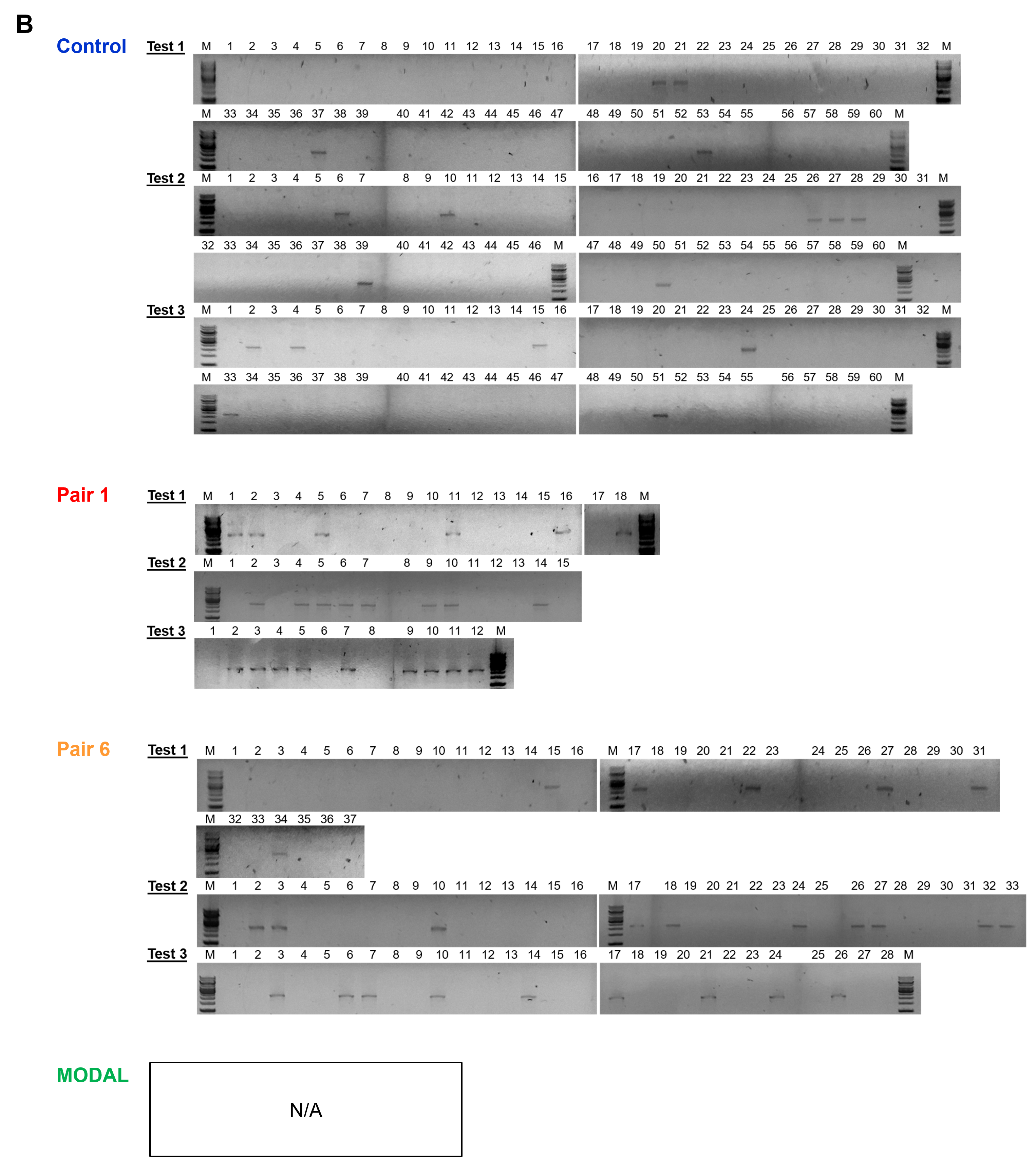


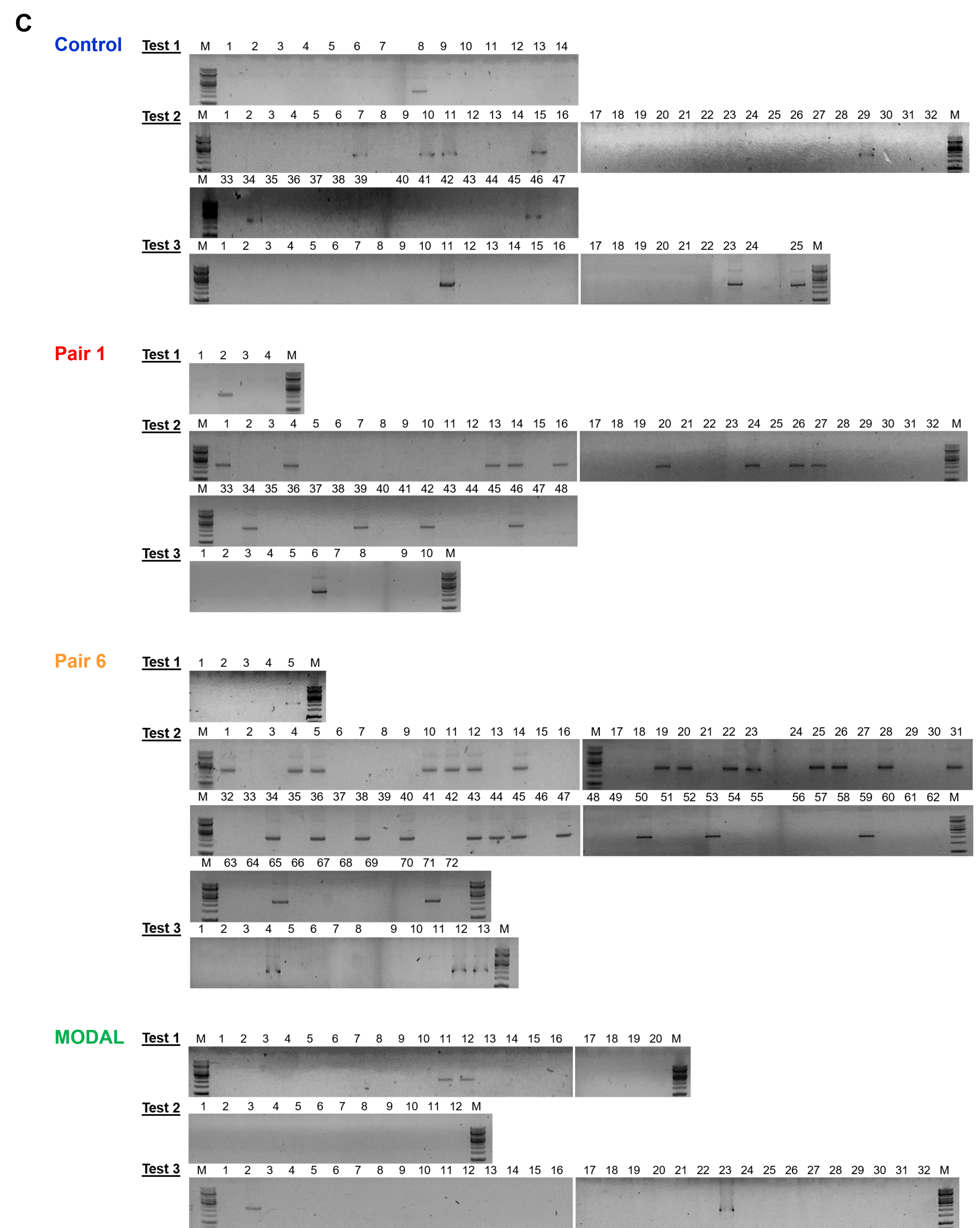


**Supplementary Figure 2.** **Gel images of PCR products.**

After conducting Gibson assembly, the colonies from each sample were checked by PCR. Gel electrophoresis was used to check the size of DNA fragments. All PCR products were applied to 1% agarose gels. The results for (A) *CDH1*, (B) *CTNNB1*, and (C) *SNAI1* were shown. N/A: not available from the samples that had no amplified homologous arms for Gibson assembly. The experiments were conducted in triplicate and labeled as test 1-3.


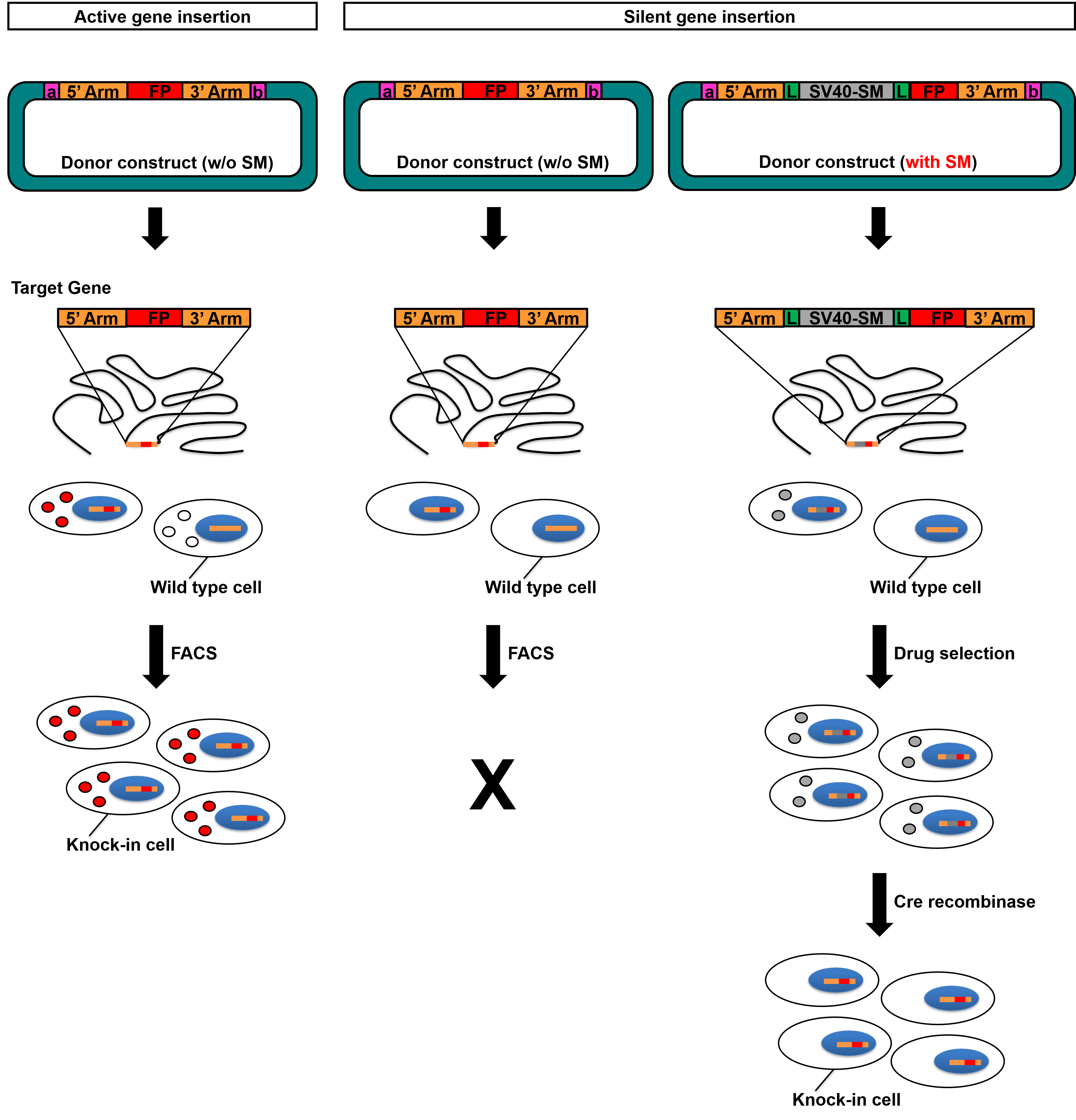


**Supplementary Figure 3.** **Layout for the CRISPR mediated FP knock-in selection.**

In general, if the knock-in target gene is an active gene, the edited cells can be selected easily through fluorescence-activated cell sorting (FACS) (left). On the contrary, for performing knock-in with a gene having low expression under normal cellular condition, the inserted FP is hardly expressed (middle). Thus, adding a removable SM (LoxP-SM-LoxP) which is driven by an external promoter, such as SV40, is necessary for picking the edited cells. Based on the expression of the SM, the cells without insertion are killed under drug treatment. Afterwards, the selected cells are treated with Cre recombinase to remove the SM (right).

**
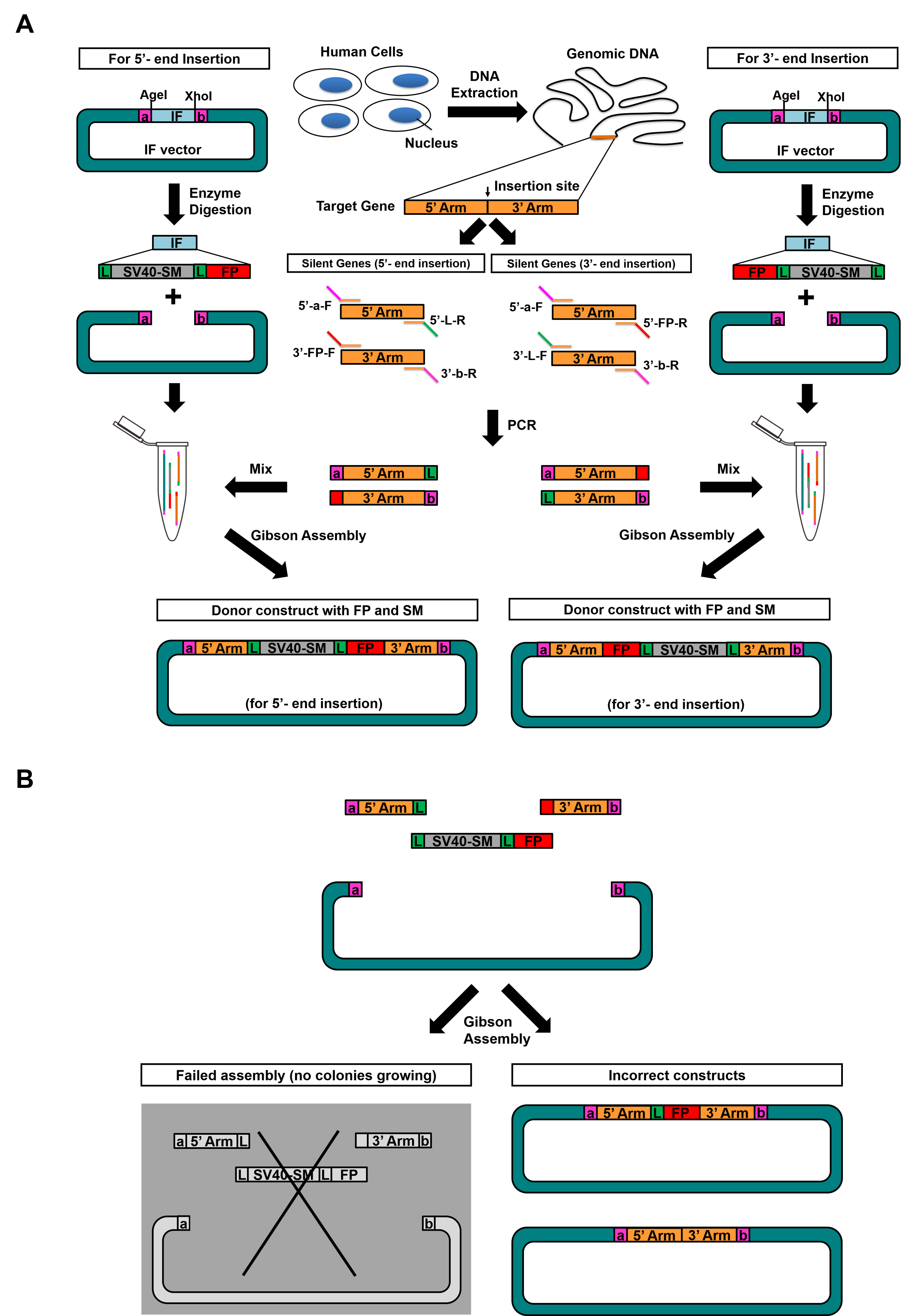
**

**Supplementary Figure 4.** **Influence of the LoxP sites on the Gibson assembly reaction.**

(A) Schematic diagram of the donor construct production for silent genes. The IF vector includes a linker pair, a FP, and a removable SM. The removable SM can be placed before (5’ end insertion, left) or after (3’ end insertion, right) the FP. After enzyme digestion, the digested IF fragment, vector, and PCR amplified products (5’/3’ arm) were mixed for Gibson assembly reaction. a: linker a, b: linker b, L: LoxP, SV40-SM: SV40 promoter and the SM. (B) The failure scenario of Gibson assembly reaction. The LoxP sites negatively influence the assembly process, thereby leading to failed assembly (left) or misassembled constructs (right).


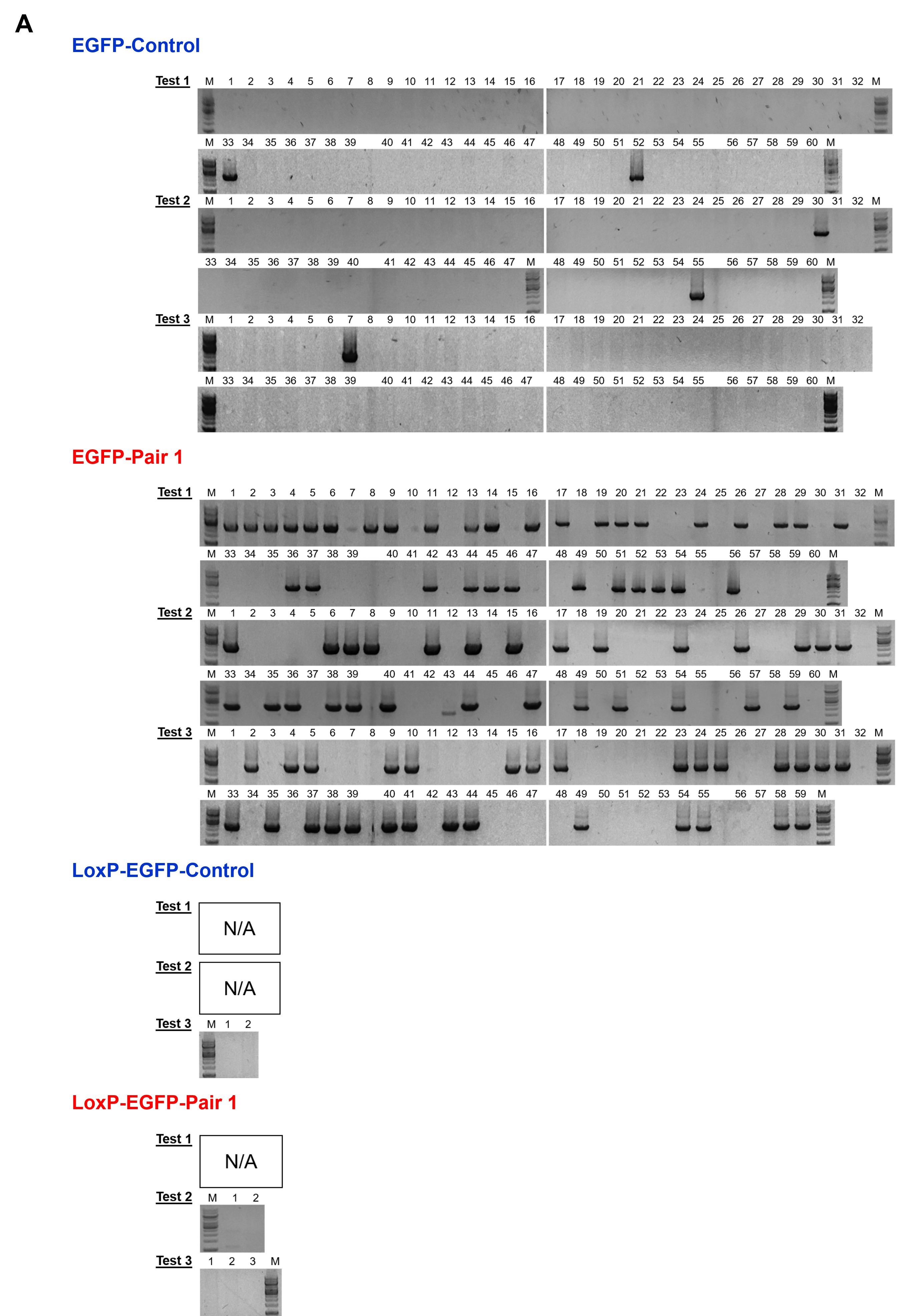


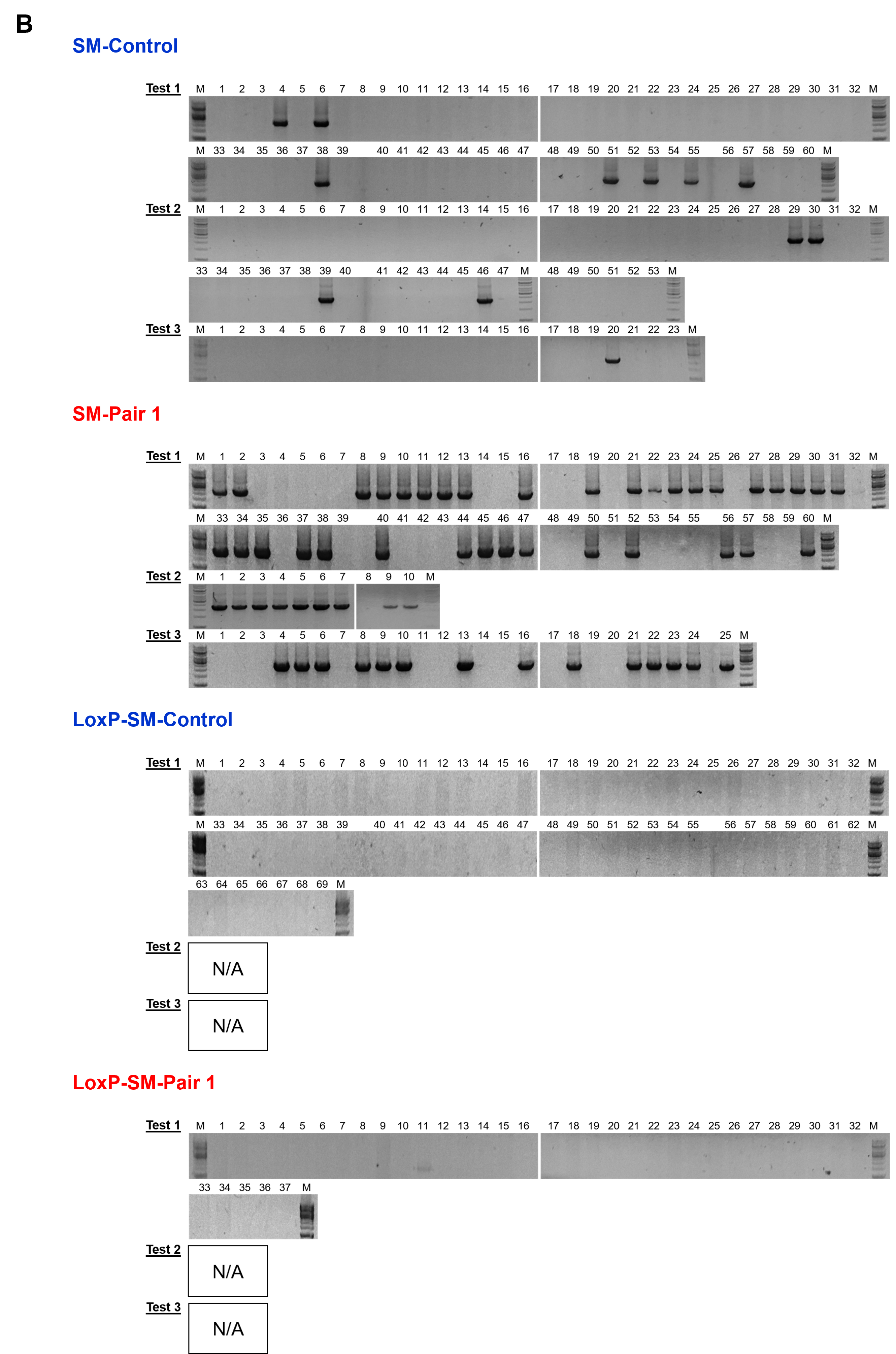


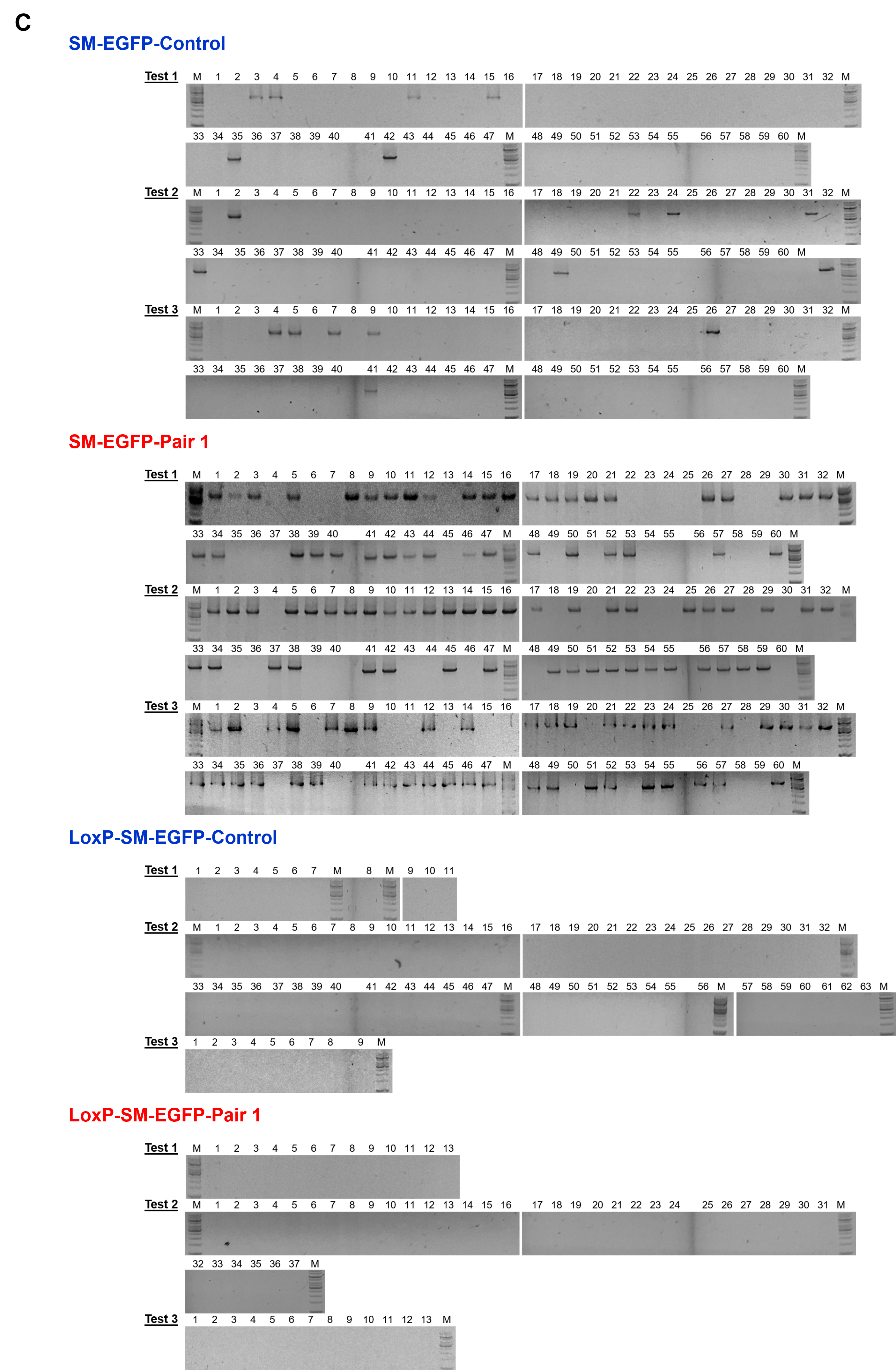


**Supplementary Figure 5.** **Gel images of PCR products.**

After conducting Gibson assembly, the colonies from each sample were checked by PCR. Gel electrophoresis was used to check the size of DNA fragments. All PCR products were applied to 1% agarose gels. The results for (A) EGFP, (B) SM, and (C) SM-EGFP were shown. N/A: not available from the samples without any colony grown on the plates. The experiments were conducted in triplicate and labeled as test 1-3.


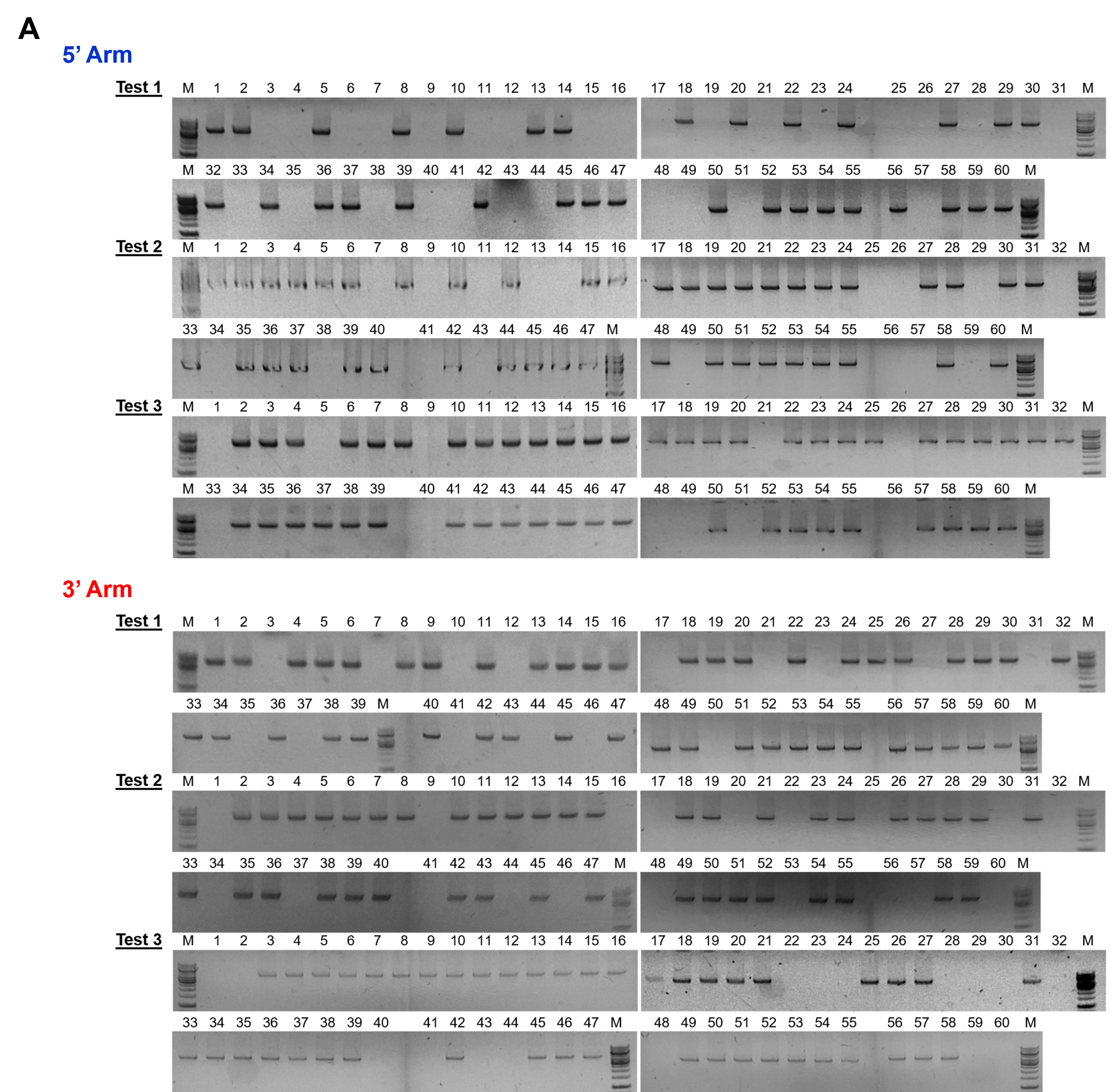


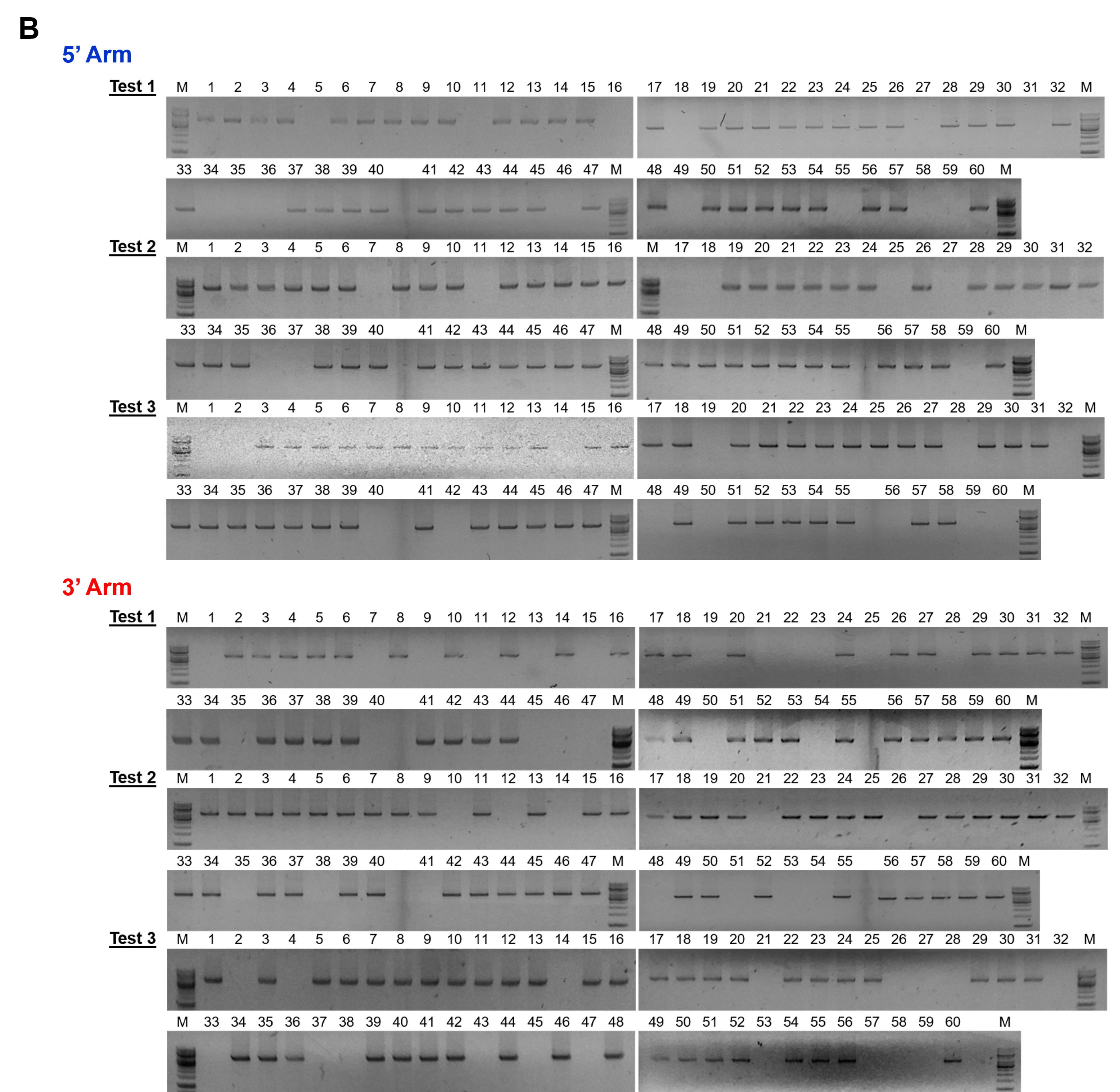


**Supplementary Figure 6.** **Gel images of PCR products.**

After conducting Gibson assembly, the colonies from each sample were checked by PCR. Gel electrophoresis was used to check the size of DNA fragments. All PCR products were applied to 1% agarose gels. The results for (A) *SNAI1* and (B) *VIM* were shown. The experiments were conducted in triplicate and labeled as test 1-3.


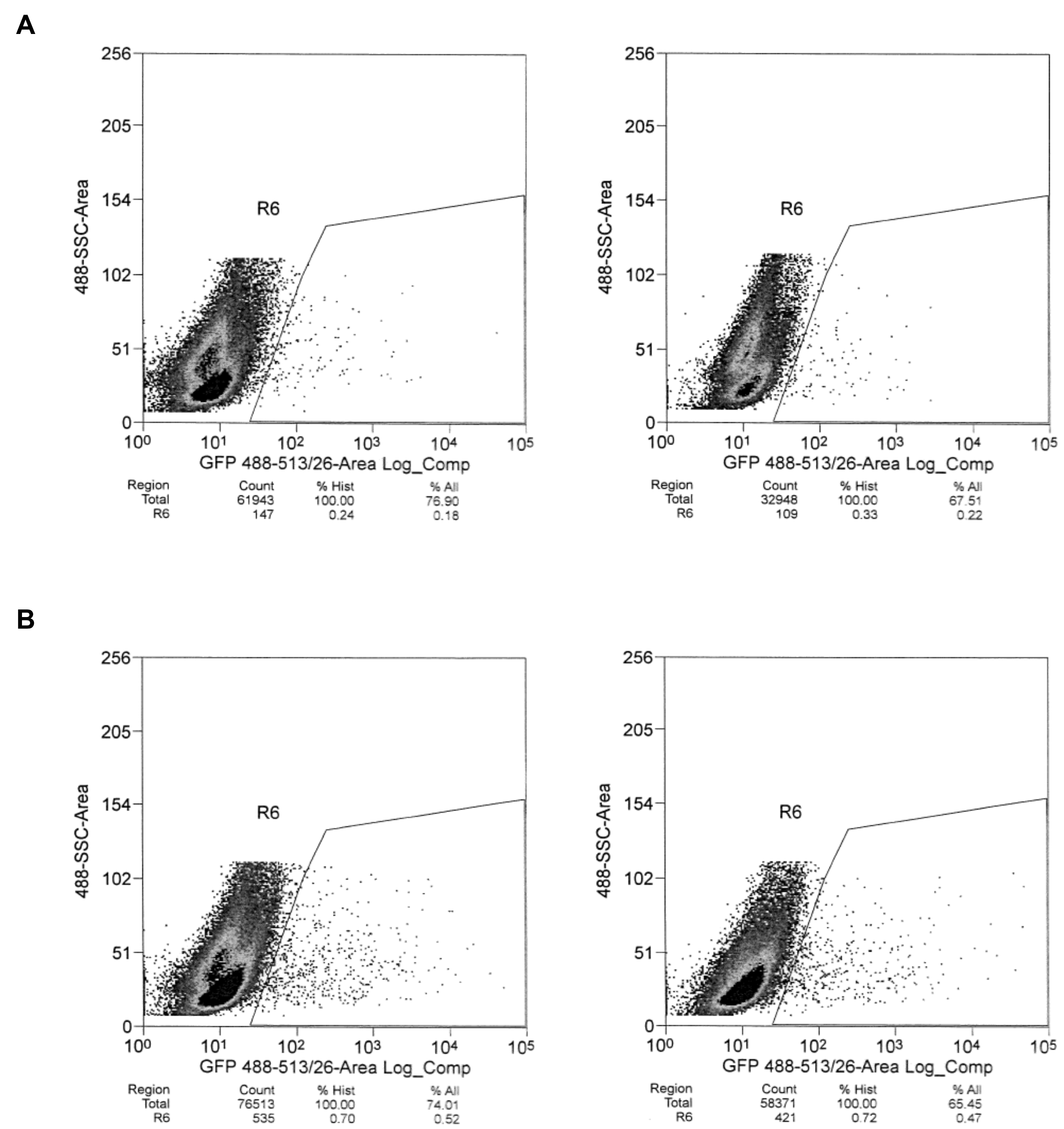


**Supplementary Figure 7.** **The results of flow cytometry for checking the efficiency of EGFP knock-in.**

Knock-in experiments were performed in T47D cells, using CDH1-EGFP and CDH1-EGFP-sgRNA as donor constructs. The successfully edited, EGFP-positive, cells were examined by flow cytometry and gated as R6. The results for CDH1-EGFP and CDH1-EGFP-sgRNA were shown in (A) and (B), respectively. The experiments were conducted in duplicate.

**
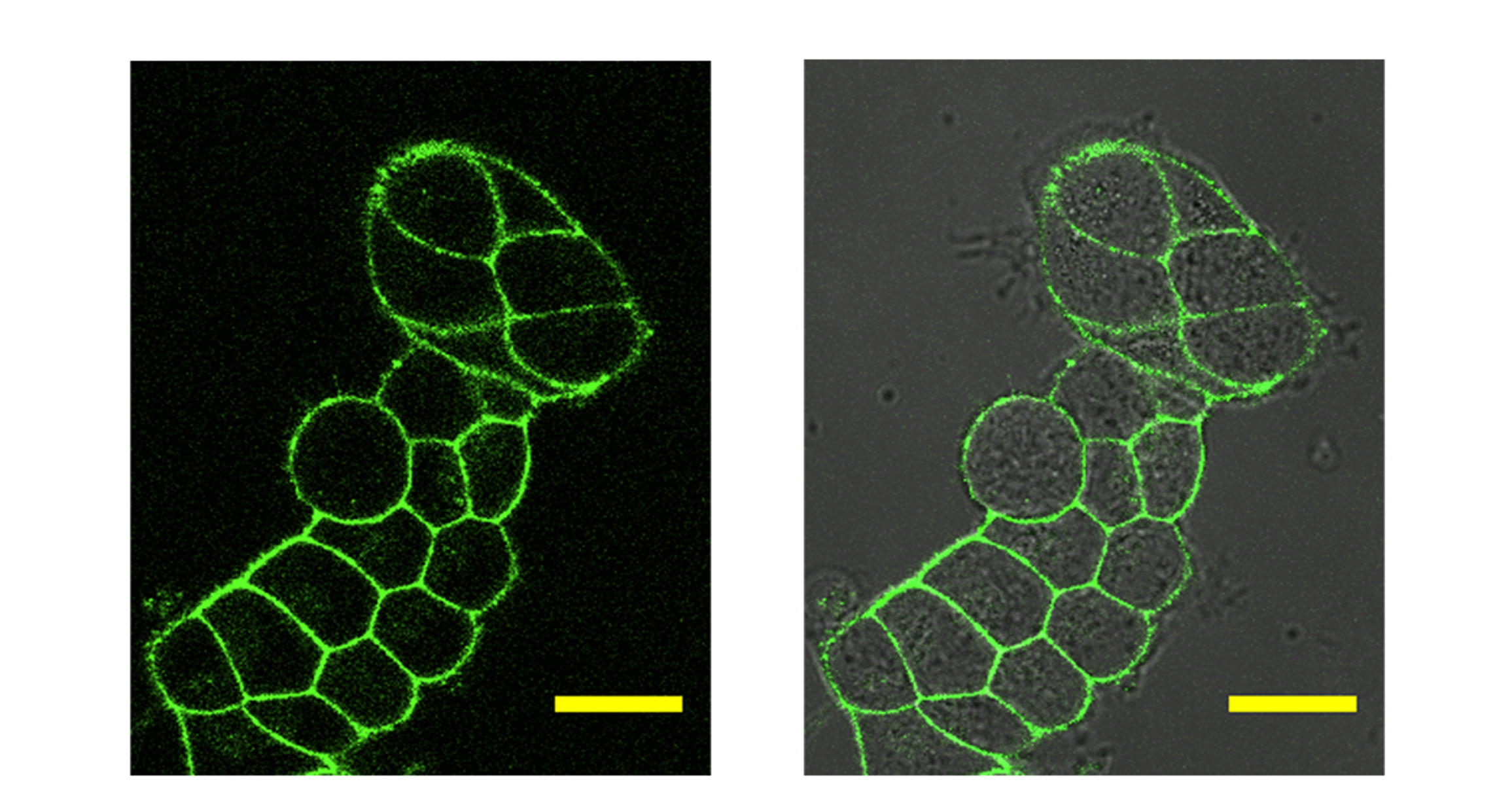
**

**Supplementary Figure 8.** **Fluorescence image of E-Cadherin*-*EGFP knock-in T47D cells.**

The image was taken under EGFP channel (left) and overlaid with the corresponding bright field image (right). Scale bar: 20 µm.

**SUPPLEMENTARY INFORMATION**

**In silico screening of the linkers.**

We generated the linker pair (a and b in Fig. 1) with a length of 30 bps for Gibson assembly reaction. Our designing principle was to build up the linkers with RESs that were between four to eight bps long. Most importantly, the RESs must have very low occurrence frequency in the human genome and the carrier vector, which is necessary for using the RESs for subsequent experiments. Among the 248 RESs identified from the human genome, we selected eight RESs that have the occurrence density less than 300 per mega base and have no existence in the carrier vector, pcDNA3, that we used in this study (see Supplementary Table 3). The chosen RESs were *EcoR*V (GATATC), *Not*I (GCGGCCG, truncated), *Xho*I (CTCGAG), *Cla*I (ATCGAT), *Hpa*I (GTTAAC), *Xba*I (TCTAGA), *Apa*I (GGGCCC), and *Age*I (ACCGGT). Then, we developed a computational pipeline (Fig. 1) to further filter the candidates. The detailed steps of the computer program are as follows:

(i) Place *Age*I at the 5’ end of linker a, and *Xho*I at the 3’ end of Linker b. These RESs were the candidates with the lowest occurrence density. Additionally, the two REs are convenient to use and less expensive compared with others, which are desirable in the two-step assembly procedure.

(ii) Generate one candidate linker pair by placing three of the remaining RESs on linker a, another three on linker b, and inserting ‘N’ between RESs or after the last RES to make the length of each linker to be 30 bps. With the same strategy, produce all possible linker pairs with various combinations and orders of the RESs as well as the inserted ‘N’s.

(iii) Calculate the sequence property for each linker pair by the Bioinformatics Toolbox in MATLAB 2016 (®MathWorks). The preferred sequences were selected based on the following criteria: (a) appropriate GC content, (b) no potential of dimerization or hairpin structures, (c) optimal salt adjusted melting temperature, (d) no GC clamp and nucleotide repeats [1].

(iv) Run the Bioinformatics Toolbox again to check cross dimerization between linker pairs and exclude the ones forming dimers.

(v) Substitute the ‘N’s in the linker pairs with ‘A’, ’T’, ’G’, or ’C’ and then repeat step 3 and 4. The pairs that meet the criteria were kept and subjected to the next step.

(vi) Perform the BLAST alignment analysis to examine the identity between the linkers and the human genome. The sequences of the remaining candidates were compared to the human genomic library and ranked according to their 100% identity scores, with the lowest ranked top 1. The 100% identity score for each linker was defined as the maximum value of the identity that the linker matched 100% to some sequence(s) in the human G+T database. In our case, the linker with lower 100% identity score was preferable (see Supplementary Fig.1).

**Manual for the linker design code**

The script was written in MATLAB and Python. In order to use the scripts, MATLAB (2016a or 2016b with Bioinformatics Toolbox), Python 2.7 and python package, Biopython **(**[**http://biopython.org/**](http://biopython.org/)**),** are required.

**1)** To run the program, an excel file as the example (Excel file named ‘parameter’) in the folder is needed. Open the file, a table with the following format is shown.

| **RES** | GATATC | GCGGCCG | CTCGAG | ATCGAT | GTTAAC | TCTAGA | GGGCCC | ACCGGT |
| --- | --- | --- | --- | --- | --- | --- | --- | --- |
| **special RES** | CTCGAG | ATCGAT | ACCGGT |  |  |  |  |  |
| **length of linker** | 30 |  |  |  |  |  |  |  |
| **number of RES in linkers** | 4 | 4 |  |  |  |  |  |  |
| **parameters for oligoprop** | 5 | 2 | 11 |  |  |  |  |  |
| **GC content threshold** | 45 | 75 |  |  |  |  |  |  |
| **melting temperature threshold** | 50 | 75.5 |  |  |  |  |  |  |
| **parameters for calculating cross dimerization** | 5 | 5.55 |  |  |  |  |  |  |
| **parameters for oligoprop** | 5 | 2 | 11 |  |  |  |  |  |
| **GC content threshold** | 45 | 60 |  |  |  |  |  |  |
| **melting temperature threshold** | 50 | 75.5 |  |  |  |  |  |  |
| **parameters for calculating cross dimerization** | 5 | 5.55 |  |  |  |  |  |  |
| **number of Blast jobs** | 1 |  |  |  |  |  |  |  |

The 1^st^ row represents for the RESs that will be included in the linkers.

The 2^nd^ row stands for the special RESs, from which two will be selected. One will be placed at the beginning of linker a and the other at the end of linker b. These RESs are also listed in the first row of the table. Leave this row blank if you do not want to have RESs at pre-determined positions.

The 3^rd^ row sets the lengths of the two linkers.

The 4^th^ row gives the number of the selected RESs in linker a and linker b, respectively. Notice that the total length of the selected RESs should be shorter than the length of the linkers.

The 5^th^ row provides the parameters for the oligoprop function which calculates the property of the sequences in Matlab. The first one is the number for minimum paired bases that form the neck of the hairpin. The second one is the number for minimum bases that form the loop of a hairpin. The third one is the number for minimum aligned bases between the sequence and its reverse. These values are the criteria for the scenario that ‘N’s are placed in the positions between the RESs.

The 6^th^ row shows the threshold for the GC content of the linkers with ‘N’ in the positions between the RESs. The first one is the lower bound and the second one is the upper bound.

The 7^th^ row defines the threshold for salt adjusted melting temperatures of the linkers with ‘N’ in the positions between the RESs. The first one is the lower bound and the second is the upper bound.

The 8^th^ row sets the parameters for calculating cross dimerization of the linker pairs with ‘N’ in the positions between the RESs. The first one is the gap open value giving the penalty for opening a gap in the alignment. The second one is the upper threshold for alignment score. A lower score means that the linker pairs have the lower chance to dimerize.

The 9^th^ row stands for the parameters as that in the 5^th^ row, while the ‘N’s in the linkers are replaced by ‘A’, ‘T’, ‘G’, or ‘C’.

The 10^th^ row gives the parameters as that in the 6^th^ row, while the ‘N’s in the linkers are replaced by ‘A’, ‘T’, ‘G’, or ‘C’.

The 11^th^ row provides the parameters as that in the 7^th^ row, while the ‘N’s in the linkers are replaced by ‘A’, ‘T’, ‘G’, or ‘C’.

The 12^th^ row shows the parameters as that in the 8^th^ row, while the ‘N’s in the linkers are replaced by ‘A’, ‘T’, ‘G’, or ‘C’.

The 13^th^ row specifies the number of files for performing BLAST alignment. The process may take long time, which depends on the speed of the internet, the server and the number of primers. Thus, we suggest dividing the BLAST job into smaller jobs. That is, when getting all the combinations of the linkers, save the linkers in several or tens of files for BLAST alignment.

**2)** Put the *parameter. xlsx* into the folder of the program.

**3)** If you have the special selected RESs, open the M file named “*run_this_file_with_special_enzyme _site*” and run it in Matlab. If you do not want to design linkers with the special selected RESs, open the M file named “*run_this_file_without_special_enzyme_site*” and run it in Matlab.

**Imaging**

The images were taken with Nikon A1+ laser scan confocal microscope (Plan Fluor 40x Oil objective, N.A. = 1.3). The channels used were DIC and GFP (excitation wavelength: 488 nm, emission wavelength: 525 nm).

**SUPPLEMENTARY PROGRAMES**

**Supplementary Program 1.** **chromo_gc_content.py**

This script is for calculating the GC content of CDS in the human genome.

**Supplementary Program 2.** **Linker design**

This package is for designing the linker pairs. Detailed information is shown in the Supplementary Information.

**REFERENCE**

1. Burpo FJ: **A critical review of PCR primer design algorithms and cross-hybridization case study.** *Biochemistry* 2001, **218**.
